# Reconstructing extrachromosomal DNA structural heterogeneity from long-read sequencing data using Decoil

**DOI:** 10.1101/gr.279123.124

**Published:** 2024-09

**Authors:** Mădălina Giurgiu, Nadine Wittstruck, Elias Rodriguez-Fos, Rocío Chamorro González, Lotte Brückner, Annabell Krienelke-Szymansky, Konstantin Helmsauer, Anne Hartebrodt, Philipp Euskirchen, Richard P. Koche, Kerstin Haase, Knut Reinert, Anton G. Henssen

**Affiliations:** 1 Department of Pediatric Oncology and Hematology, Charité–Universitätsmedizin Berlin, 13353 Berlin, Germany;; 2 Experimental and Clinical Research Center of the Max Delbrück Center and Charité Berlin, 13125 Berlin, Germany;; 3 Charité–Universitätsmedizin Berlin, 10117 Berlin, Germany;; 4 Freie Universität Berlin, 14195 Berlin, Germany;; 5 Max Delbrück Center for Molecular Medicine, 13125 Berlin, Germany;; 6 Friedrich-Alexander-Universität Erlangen-Nürnberg, 91054 Erlangen, Germany;; 7 German Cancer Consortium (DKTK), partner site Berlin, a partnership between DKFZ and Charité–Universitätsmedizin Berlin, 10117 Berlin, Germany;; 8 Department of Neuropathology, Charité–Universitätsmedizin Berlin, corporate member of Freie Universität Berlin and Humboldt-Universität zu Berlin, 13353 Berlin, Germany;; 9 Center for Epigenetics Research, Memorial Sloan Kettering Cancer Center, New York, New York 10065, USA

## Abstract

Circular extrachromosomal DNA (ecDNA) is a form of oncogene amplification found across cancer types and associated with poor outcome in patients. ecDNA can be structurally complex and can contain rearranged DNA sequences derived from multiple chromosome locations. As the structure of ecDNA can impact oncogene regulation and may indicate mechanisms of its formation, disentangling it at high resolution from sequencing data is essential. Even though methods have been developed to identify and reconstruct ecDNA in cancer genome sequencing, it remains challenging to resolve complex ecDNA structures, in particular amplicons with shared genomic footprints. We here introduce Decoil, a computational method that combines a breakpoint-graph approach with *LASSO* regression to reconstruct complex ecDNA and deconvolve co-occurring ecDNA elements with overlapping genomic footprints from long-read nanopore sequencing. Decoil outperforms de novo assembly and alignment-based methods in simulated long-read sequencing data for both simple and complex ecDNAs. Applying Decoil on whole-genome sequencing data uncovered different ecDNA topologies and explored ecDNA structure heterogeneity in neuroblastoma tumors and cell lines, indicating that this method may improve ecDNA structural analyses in cancer.

Circular extrachromosomal DNA (ecDNA) is an important form of oncogene amplification in cancer (Kim et al. 2020), which can be formed through multiple mechanisms (Storlazzi et al. 2006; Shoshani et al. 2021; Yi et al. 2022) and have a large size (up to several megabases) (Pecorino et al. 2022). As a result, ecDNA can be structurally diverse, with different functional outcomes. The structure of ecDNA can impact gene regulation through the rearrangement of regulatory elements as well as topologically associated domain (TAD) boundaries (Helmsauer et al. 2020). To explore ecDNA diversity and complexity, high-resolution computational methods to reconstruct ecDNA with high accuracy from genome sequencing data are required. The reconstruction of ecDNA from sequencing data remains challenging owing to the variable complexity and intratumor heterogeneity of these circular elements. On the one hand, a single ecDNA can be heavily rearranged and contain low-complexity sequence regions (e.g., repeats), which pose a challenge to mapping and de novo assembly-based methods. On the other hand, one tumor can contain different ecDNA elements (Hung et al. 2022; Chamorro González et al. 2023), which can originate from either different or shared genomic locations (Verhaak et al. 2019). The latter scenario may be very challenging for ecDNA reconstruction, as different co-occurring ecDNA elements have overlapping genomic footprints, making it difficult to attribute the overlapping features to each of the different circular elements. In the past years, several computational tools have been developed to reconstruct ecDNA from different input data. Some methods were developed to detect circularized DNA regions by identifying the breakpoints leading to circularization (circle-enrich-filter [Koche et al. 2020], Circle-Map [Prada-Luengo et al. 2019], ecc_finder [Zhang et al. 2021]). These approaches are suitable for detecting simple circular amplicons, but they overlook complex ecDNA structures. To overcome these limitations, more recently, methods focused on reconstructing complex ecDNA based on different technologies, for example, short-read whole-genome sequencing (AmpliconArchitect) (Deshpande et al. 2019), optical-mapping combined with short-read sequencing (AmpliconReconstructor) (Luebeck et al. 2020), and long-read sequencing, were developed (CReSIL) (Wanchai et al. 2022). Lastly, methods have been developed to delineate ecDNA structural heterogeneity (Hung et al. 2022) by isolating and reconstructing individual ecDNA elements, leveraging a priori knowledge about the ecDNA present in the sample of interest. However, a method that reconstructs complex ecDNA structures and captures heterogeneity by distinguishing between ecDNA elements with overlapping genomic footprints from whole-genome sequencing (WGS) data without such a priori knowledge is still largely missing to date. We here present deconvolve extrachromosomal circular DNA isoforms from long-read data (Decoil), a computational method to reconstruct genome-wide complex ecDNA elements and deconvolve individual ecDNAs with shared genomic sequences from bulk whole-genome long-read sequencing using Nanopore technology. Decoil is a graph-based approach integrating the structural variant (SV) and coverage profiles to deconvolve and reconstruct complex ecDNAs. It uses *LASSO* regression to infer likely ecDNA structures and estimate their relative proportions by accounting for circular elements with overlapping genomic footprints. The model can separate and reconstruct individual ecDNA elements with shared genomic regions, which is not possible by previously published methods. Decoil may improve the resolution to study ecDNA structural intra/inter-tumor heterogeneity from bulk sequencing data.

## Results

### An overview of the Decoil algorithm

**Figure 1.**
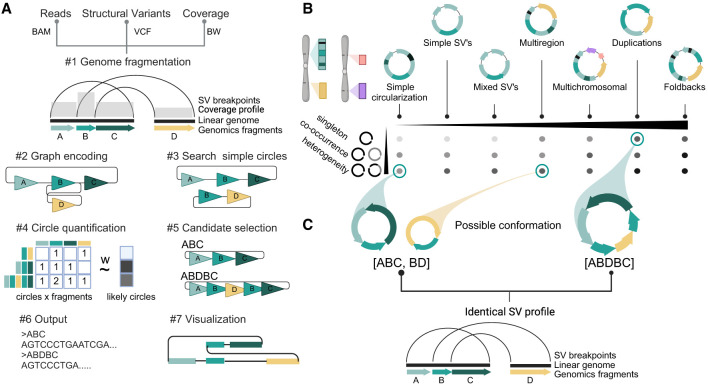
Decoil algorithm overview and an ecDNA ranking system based on its structural diversity. (*A*) Schematic of the Decoil algorithm depicting the major steps: (#1) genome fragmentation, (#2) graph encoding, (#3) search simple circles, (#4) circle quantification, (#5) candidate selection, (#6) output, and (#7) visualization. Step #7, visualization, is performed by the Decoil-viz module (see Methods). (*B*) ecDNA diversity. The *x*-axis displays the seven ecDNA topologies (e.g., simple circularization, multiregion, multichromosomal) with increasing computational complexity as defined in this paper. The *y*-axis displays different scenarios of ecDNA composition per sample, that is, singleton (presence of a single ecDNA structure), co-occurrence (presence of different ecDNA species, with nonoverlapping genomic regions), and heterogeneity (presence of different ecDNA species, with overlapping genomic regions). The gradient matrix depicts schematically the ecDNA reconstruction difficulty levels for the different scenarios (*y*-axis) and topologies (*x*-axis), which are addressed by Decoil algorithm. Light gray means low difficulty; black, increased difficulty. (*C*) Computational challenge formulation. The *left* panel displays a heterogeneity scenario, in which two different ecDNA elements (ABC, BD) share the genomic footprint (B fragment); the *right* panel displays a single large structure (ABDBC) containing interspersed-duplication rearrangement (B fragment duplicated on ecDNA). Both scenarios lead to the same SV breakpoint profile. To infer the likely conformation, we perform step #4 in *A*. Created with BioRender (https://www.biorender.com/).

Decoil reconstructs complex ecDNA structures from bulk long-read nanopore sequencing data using aligned sequencing reads, SVs, and coverage profiles as input (Fig. 1A). The genome is initially fragmented using a clean breakpoint set (Fig. 1A#1). A weighted undirected multigraph is built to encode the structural rearrangements, in which nodes are objects that represent the genomic nonoverlapping fragments and edges represent the SVs (Fig. 1A#2; Supplemental Fig. S11).

**Figure 2.**
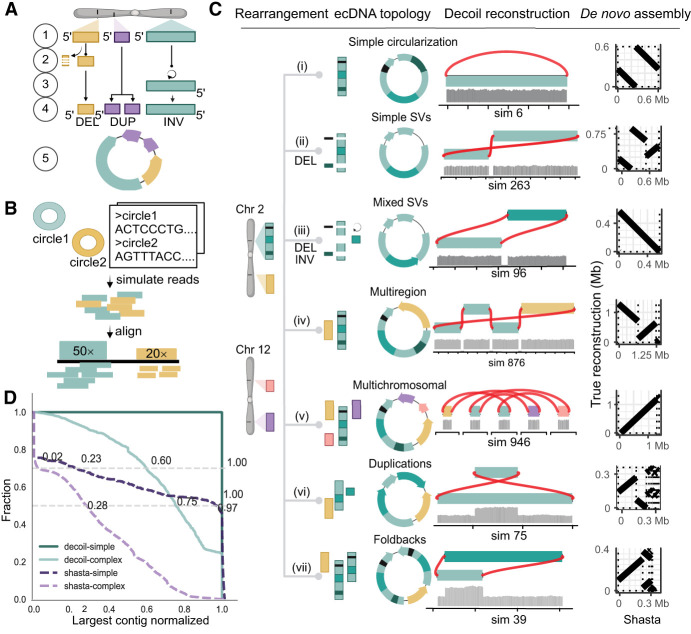
Decoil reconstructs complex ecDNA elements with high fidelity from simulated data. (*A*) Simulation strategy for generating individual ecDNA templates consists of the following steps: (*1*) choose genomic position, (*2*) simulate small deletions (DELs), (*3*) simulate inversion (INV), (*4*) simulate tandem-duplication (DUP), and (*5*) generate DNA sequence template (FASTA). The example depicts an ecDNA template harboring 1 × DEL (yellow), 1 × DUP (purple), and 1 × INV (green). (*B*) Pipeline for generating in silico long reads based on one or more ecDNA templates, at different depths of coverage. (*C*) The ecDNA topologies. Examples of ecDNA reconstructions performed by Decoil for simulated ecDNA elements, for the seven different topologies. The gray track represents the coverage of the aligned reads. The *right* column shows the Shasta de novo assembly (*x*-axis) against the true structure (*y*-axis). (*D*) Decoil and Shasta assembly contiguity for simple (*i–v*) and complex topologies (*vi*,*vii*). The *y*-axis represents the fraction of reconstructions with a specific contiguity (*x*-axis). The *x*-axis represents the larger contig normalized by the true structure length. One indicates a good reconstruction, zero poor reconstruction. Values greater than one refer to reconstructions larger than the true structure. The gray horizontal lines are at the 0.5 and 0.7 fractions.

Next, the graph is explored using a depth-first search approach to discover genome-wide simple circular paths (Fig. 1A#3; Supplemental Fig. S10A–C). These paths can represent a standalone circular element or be a subcomponent of a more complex circular structure that is represented in the graph as a series of nested simple circular paths. Subsequently, to address this challenge, simple circular paths with at least one overlapping genomic fragment are merged into a derived larger circular structure (Supplemental Fig. S10C). To avoid exponential growth for the cycles merge, only cycles sufficiently dissimilar are candidates to be considered (see Methods). This allows us to capture heavily rearranged circular structures and to discover large duplications.

**Figure 3.**
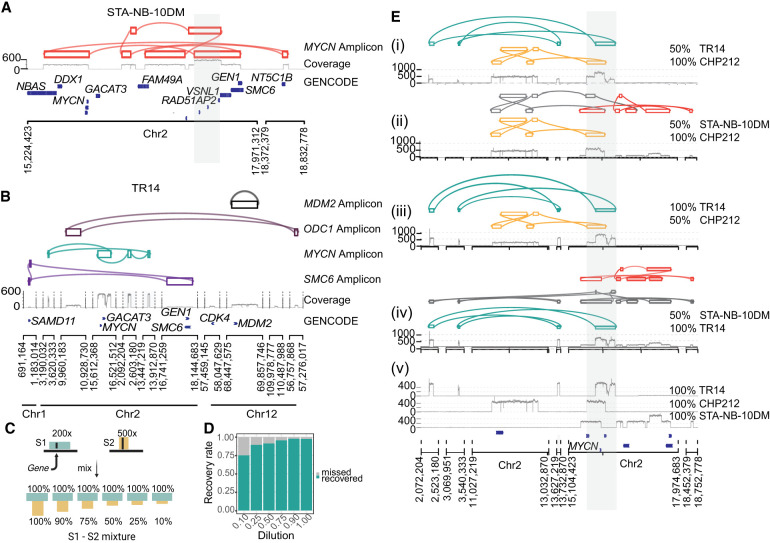
Decoil captures the ecDNA structure complexity and heterogeneity in neuroblastoma cell lines. (*A*) STA-NB-10DM ecDNA reconstruction by Decoil (*top*), coverage track (*middle*) of the aligned reads to reference genome GRCh38/hg38, and GENCODE V42 annotation (*bottom*). The gray highlighted region Chr 2: 17,221,081–17,538,185 (GRCh38/hg38) represents an interspersed duplication. (*B*) ecDNA elements co-occurrence reconstructed by Decoil in TR14 (*top* four tracks), the coverage track (*middle*), and GENCODE V42 annotation (*bottom*). (*C*) In silico dilution strategy, in which two samples, S1 (green) and S2 (yellow), are mixed at different ratios to generate mixture of ecDNAs that overlap in the genomic space. (*D*) ecDNA breakpoint recall (*y*-axis) for the in silico cell line mixtures, split by the dilution ratio (*x*-axis). An ecDNA element harboring *MYCN* is present in every one of the three cell lines, that is, CHP212, TR14, and STA-NB-10DM, and is composed of 10, eight, and 14 breakpoints, respectively. The other co-occurring ecDNA elements in TR14 are also added to the analysis and have four (*ODC1*-), two (*MDM2*-), and six (*SMC6*-amplicon) breakpoints. (*E*) ecDNA reconstruction visualization using Decoil-viz for the in silico ecDNA mixtures. (*i*–*iv*) The reconstructed ecDNA structures by Decoil in cell line mixtures (green, TR14; yellow, CHP212; and orange, STA-NB-10DM) overlap in the genomic space at the *MYCN* locus (gray highlight). (*v*) Coverage track for pure (100%) TR14, CHP212, and STA-NB-10DM cell lines. Misassemblies are depicted in gray.

To identify the likely ecDNA elements present in the sample, all simple and derived circle candidates are leveraged as features to fit a *LASSO* regression against the mean coverage profile of the aligned reads (Fig. 1A#4). The model (1) selects the likely circles explaining the amplification and (2) estimates their proportions within the sample (Supplemental Fig. S10D–F). This approach enables reconstruction of the ecDNA elements with overlapping genomic regions, which is difficult to resolve computationally (Fig. 1B,C). This makes Decoil a versatile tool to characterize intratumor ecDNA heterogeneity.

**Figure 4.**
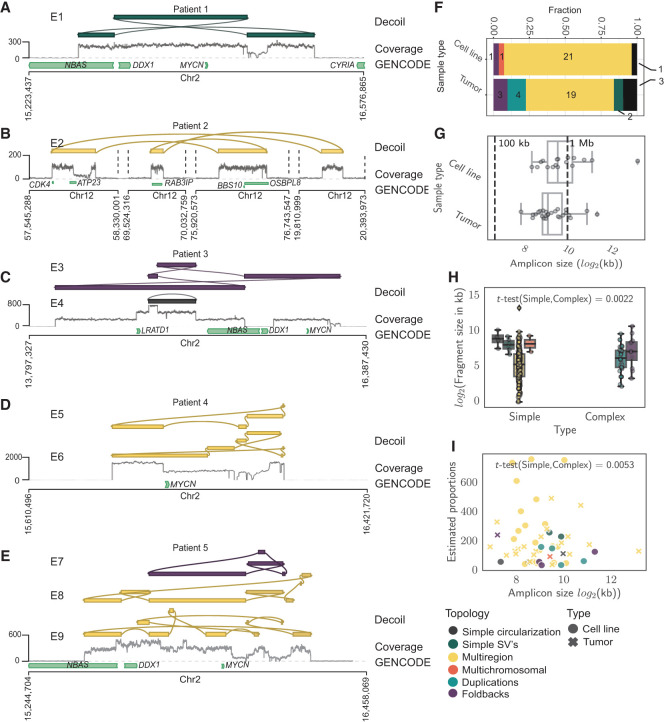
Decoil recovers structurally complex ecDNA elements in primary cancers. Examples of ecDNA structure reconstruction of Simple SVs (*A*), Multiregions (*B*,*D*), Foldbacks (*C*), and Duplications/Foldbacks topologies (*E*) in patient samples. (*A*–*E*) The tracks represent the Decoil reconstruction (*top*), coverage of the aligned Nanopore reads to reference genome GRCh38/hg38 (*middle*), and GENCODE V42 annotation (*bottom*). The top three reconstructions were included if labeled as ecDNA and had estimated proportions 30 or more copies (*A*–*E*). E1–E9 are the IDs for each reconstruction (Supplemental Table S4). (*F*) The topology spectrum of the reconstructed ecDNA structures by Decoil for the five cell lines and nine patient samples. (*G*) ecDNA reconstruction total size (*x*-axis) distribution (*y*-axis) for all data (five cell lines, nine primary tumor samples). (*H*) ecDNA fragment size distribution split for simple (Simple circularization, Simple SVs, Multiregion, Multichromosomal) or complex (Duplications, Foldbacks) topologies. (*I*) ecDNA reconstruction total size (*x*-axis) against estimated proportions (*y*-axis) computed by Decoil. (*H,I*) *t*-Test statistics were applied to test the significance of the ecDNA proportions between simple and complex topologies. All reconstructions labeled as ecDNA and with estimated proportions of 30 or more copies were included in panels *F*–*I*. Box plot shows Q1 (25%), Q2 (median), Q3 (75%), and interquartile range IQR = Q3–Q1; whiskers are 1.5 × IQR. The colors in *A*–*F*,*H* correspond to the legend in *I*.

Lastly, a filtered confident set of circular paths is generated (Fig. 1A#5), together with the annotated topology (as defined below), proportion estimates (Fig. 1A#6), and reconstruction thread visualization by the Decoil-viz module (Fig. 1A#7).

### Ranking and simulating ecDNA topologies to capture ecDNA structure diversity

ecDNA structures are complex rearrangements, and currently, no guidelines or gold-standard data sets exist to assess the quality and performance of the reconstructions computed by algorithms, as exists for the detection of single-nucleotide variants (SNVs), insertion–deletions (indels), and SVs (Olson et al. 2022, 2023). This makes the evaluation of the Decoil algorithm contingent on high-quality simulated data. To capture a diverse spectrum of ecDNA elements, the SV profile obtained from the read alignment was used as information to systematically rank the ecDNA structure by computational complexity (Fig. 1B). Thus, based on the different SV combinations present on the ecDNA element, we propose seven ecDNA topologies (Fig. 2): i. Simple circularization, ii. Simple SVs, iii. Mixed SVs, iv. Multiregion, v. Multichromosomal, vi. Duplications, and vii. Foldbacks. These ecDNA topologies were leveraged to simulate rearrangements on the amplicon in order to create a representative and comprehensive collection of more than 2000 ecDNA templates (Fig. 2A), based on which we generated in silico long reads at different depths of coverage. This collection serves as a benchmark data set for evaluating Decoil's reconstruction performance across varying computational complexities and could be a useful data set for future ecDNA genomic studies.

### Decoil's performance evaluation to reconstruct ecDNA elements from simulated data

The accuracy of ecDNA reconstructions was quantified using the normalized largest contig as a score to measure the assembly contiguity (see section “Performance evaluation on simulated data” in the Methods). Decoil reconstructed simple ecDNA topologies with high fidelity from simulated data, that is, topologies i–v (more than 700 simulations) (Fig. 2C,D). For the complex topologies, that is, vi and vii, Decoil reconstructed correctly at least 60% of the true structure (largest contig normalized > 0.6) (Fig. 2D) in >70% of the simulations (more than 1900 simulations). Poorly resolved ecDNA elements (largest contig normalized < 0.6) often contained mixed rearrangements including nested duplications and foldbacks, suggesting that such ecDNA elements are more challenging to reconstruct. To demonstrate the utility and feasibility of the method, Decoil was compared against Shasta (Shafin et al. 2020), a de novo assembler, and CReSIL (Supplemental Fig. S7; Supplemental Table S2; Wanchai et al. 2022) using different QUAST metrics (e.g., largest contig, largest alignment, auN), as described in the Supplemental Methods. CReSIL reconstructs a continuous full alignment for >65% of simple topologies with high fidelity (Supplemental Fig. S7E). Decoil outperformed Shasta and CReSIL for both simple and complex topologies in terms of sequence contiguity and completeness (Supplemental Table S1).

### Decoil recapitulates ecDNA complexity and their co-occurrence in well-characterized cancer cell lines

To show the versatility of the algorithm, Decoil was applied to shallow whole-genome nanopore sequencing of three neuroblastoma cell lines, namely, CHP212, STA-NB-10DM, and TR14, for which ecDNA elements were previously characterized based on various circular DNA enrichment methods and/or validated using fluorescence in situ hybridization (FISH) (Storlazzi et al. 2010; Helmsauer et al. 2020; Hung et al. 2021). Decoil's reconstructions recapitulated the previously validated ecDNA element in CHP212 with high fidelity (Supplemental Fig. S1A,B). An ecDNA harboring *MYCN* and a gene fusion between *SMC6* and *FAM49A* was previously observed in STA-NB-10DM cells (Storlazzi et al. 2010), which was confirmed by Decoil's reconstruction (Fig. 3A). The ecDNA element in STA-NB-10DM was predicted to be 2.1 Mb in size, with an estimated proportion of 171 amplicon copies, harboring an interspersed duplication according to Decoil reconstruction (Fig. 3A). Multiple co-occurring ecDNA elements, referred to as ecDNA species in a previous report, were observed in TR14 cells (Hung et al. 2021). The three different ecDNA elements, containing *MYCN*, *ODC1*, and *MDM2*, were reconstructed by Decoil with high fidelity in TR14 (Fig. 3B). Additionally, Decoil identified a previously unreported 1.09 Mb (Supplemental Table S3), multichromosomal ecDNA element containing fragments from Chromosome 1 and Chromosome 2, with an estimated proportion of 20 amplicon copies, harboring *SMC6* and *GEN1* (Fig. 3B). This is the largest amplicon and has the lowest number of estimated copies relative to the other co-occurring ecDNA elements, which may be the reason why other reports have not been able to identify it so far. For comparison, the reconstruction's contiguity in the cell lines was evaluated also using Shasta. For CHP212, the agreement between Decoil and Shasta was 100% (Supplemental Fig. S1B,C). In STA-NB-10DM, the interspersed duplication on the ecDNA indicates increasing reconstruction complexity. Thus, Shasta did not assemble a contiguous circular element (Supplemental Fig. S2A), whereas Decoil identified a contiguous circular path through the graph of this ecDNA element (Fig. 3A). For TR14, the structures of amplicons harboring *SMC6*, *MDM2*, or *ODC1* were consistent between Decoil and Shasta (Supplemental Figs. S3, S2B). Additionally, the *MYCN*-containing ecDNA was reconstructed by Decoil (Fig. 2B; Supplemental Fig. S4A) but was not fully resolved by Shasta (Supplemental Fig. S4B) owing to overlapping rearrangements at the *MYCN* locus (Supplemental Fig. S2B). Thus, Decoil is a versatile algorithm to (1) reconstruct complex ecDNA elements in cancer cell lines and (2) discover previously unknown ecDNAs from long-read sequencing data.

### Decoil can recover ecDNA structure heterogeneity

To demonstrate that Decoil can resolve structurally distinct ecDNA elements with an overlapping genomic footprint, we generated 33 in silico mixtures, by pair-wise combination of three neuroblastoma cell lines at different ratios, namely, CHP212, STA-NB-10DM, and TR14, each containing a structurally distinct ecDNA element harboring a *MYCN* gene (see section “Evaluate amplicon's breakpoint recovery in ecDNA mixtures” in the Methods) (Fig. 3C,E). In the 50%–100% mixtures (Fig. 3E), TR14 and CHP212 *MYCN*-amplicons were accurately resolved by Decoil (Fig. 3E, i–iv) even though they share genomic regions with the other co-occurring ecDNA elements in the mixture. The STA-NB-10DM *MYCN*-amplicon was partially reconstructed in mixtures (Fig. 3E, ii,iv). Overall, the breakpoint junctions of the individual ecDNA elements were recovered in the different mixtures with a recall of 93% (Fig. 3D). These results suggest that Decoil can distinguish between different co-occurring ecDNA elements with overlapping genomic footprints, enabling the measurement of structural ecDNA heterogeneity.

### Exploring structural ecDNA complexity in cancer patients using Decoil

To explore structural ecDNA complexity in tumors, shallow whole-genome nanopore sequencing on a cohort of 13 neuroblastomas was performed, of which 10 harbored at least one ecDNA (experimentally confirmed by FISH) and three negative controls (no ecDNA present). Decoil did not detect any ecDNA in the negative control cohort and reconstructed at least one amplicon for the other nine samples, with genomic fragments originated from Chromosome 2 or Chromosome 12. The reconstructed ecDNA elements varied greatly in their complexity (Fig. 4F; Supplemental Table S4) and ranged from very simple (Fig. 4A) or multiregion (Fig. 4B) to heavily rearranged complex structures (Fig. 4C–E). Decoil reconstructs for patient 4's two ecDNA elements with individual estimated proportions of more than 700× (Supplemental Table S4), resolving the same breakpoints as previously published (Chamorro González et al. 2023). For some patients, Decoil reconstructed multiple circular elements with different estimated relative proportions, which suggests ecDNA structural heterogeneity (Fig. 4E). Multiregion topology seemed to be the most frequent ecDNA topology identified in patients, consistent with the ecDNA elements detected in cell lines (Fig. 4F). Decoil reconstructed ecDNA elements with a mean size of 1.4 Mb in cell lines and 0.7 Mb in patient samples (Fig. 4G; Supplemental Table S4), in line with other studies (Pecorino et al. 2022). Contiguous genomic fragments on ecDNA had a mean size of 138 kb in cell lines and 121 kb in patient samples (Supplemental Fig. S5B). Although the ecDNA size was conserved for the different topologies (Supplemental Fig. S5A), complex ecDNA elements had significantly shorter fragments than did simple ecDNAs (Fig. 4H; Supplemental Fig. S5C). Lastly, simple ecDNA had higher copy numbers than complex ones in this cohort (Fig. 4I; Supplemental Fig. S5D) and may indicate yet-unknown structural features that may influence ecDNA maintenance and/or oncogene regulation.

### Memory and runtime

Using the simulated (∼0.01× WGS mean coverage) and real data set (3–7× WGS mean coverage), we showed that Decoil is more efficient in terms of runtime and memory compared with CReSIL. Decoil standalone runs in <5 min (median) for both simulated and real data sets (Supplemental Fig. S8). Decoil-pipeline requires a median of <1 h for a 4× thread parallelization, which is 8× faster than CReSIL. The maximum memory usage (MaxRss) for the real data set by Decoil standalone and Decoil-pipeline was <4 GB compared with 15 GB and 192 GB for CReSIL and Shasta, respectively (median values) (Supplemental Table S5).

## Discussion

The structural complexity and heterogeneity of ecDNA make its reconstruction from sequencing data a challenging computational problem. We here presented Decoil, a method to reconstruct co-occurring complex ecDNA elements.

Because of their random mitotic segregation, many ecDNA elements, which may structurally differ, co-occur in the same cancer cells (Chamorro González et al. 2023). Disentangling ecDNA with shared genomic regions has not yet been addressed by other methods, and it cannot be resolved by de novo assemblers (e.g., Shasta) when sequencing reads are smaller than the size of genomic fragments (mean length > 125 kb in our cohort) within an ecDNA element. Decoil uses *LASSO* regression to reconstruct distinct ecDNA elements with overlapping genomic footprints, which enables the exploration of ecDNA structural heterogeneity. We have chosen this approach as it performed reasonably in our hands compared with other linear regression models (Supplemental Fig. S6). One limitation of our methods represents the correct decomposition into distinct ecDNA elements for structures containing repetitive regions. This would lead to incomplete structural resolution; for example, the order of the repeat-containing genomic segments might remain ambiguous. Furthermore, ecDNA present at low abundance or SVs not detected owing to computational limits may affect Decoil's performance. Measuring the limit of detection of Decoil was not addressed in this paper, as it will require comprehensive tumor data sets with validated ecDNA structures. Ultra-long-read sequencing (>100 kb) at high coverage, or other sequencing technologies, may improve the SV detection and structural resolution of ecDNA using Decoil, but the aforementioned scenarios may remain difficult to resolve.

A structure–function relationship was first demonstrated for ecDNA by reports describing regulatory elements on ecDNA (Morton et al. 2019; Helmsauer et al. 2020; Koche et al. 2020; Hung et al. 2021). These reports revealed that complex ecDNAs rewire tissue-specific enhancer elements to sustain high oncogene expression (Wu et al. 2019; Helmsauer et al. 2020). This also occurs through formation of new TADs (Helmsauer et al. 2020). Decoil was able to identify multiregion ecDNA elements, which were previously linked to enhancer hijacking (Helmsauer et al. 2020), suggesting that it may help map such alterations in cancer. We envision that combining Decoil with DNA methylation analysis from the same nanopore sequencing reads may enable exploration of potential regulatory heterogeneity in co-occurring ecDNA elements, which was not previously possible.

The reconstruction of ecDNA in a cohort of neuroblastoma tumors and cell lines using Decoil suggested that structurally simple ecDNA elements occurred at higher copy numbers and were larger in size compared with complex ecDNA. This might be because of computational biases, as complex structures are more difficult to reconstruct, and certainly needs to be verified in larger tumor cohorts. However, it is reasonable to speculate that ecDNA complexity could influence ecDNA maintenance or impact its copy number in as-yet-unidentified ways. Future analyses using Decoil may help verify this observation and address such questions.

In summary, we envision that Decoil will advance the exploration of ecDNA structural heterogeneity in cancer and beyond, which is essential to better understand mechanisms of ecDNA formation and its structural evolution, and may serve as the basis to identify DNA elements required for oncogene regulation and ecDNA maintenance.

## Methods

### Decoil algorithm

Decoil (deconvolve extrachromosomal circular DNA isoforms from long-read data) is a graph-based method to reconstruct circular DNA variants from shallow long-read WGS data. This uses (1) SVs and (2) focal amplification information to reconstruct circular ecDNA elements. The algorithm consists of seven modules: *Genome fragmentation*, *Graph encoding*, *Search simple circles*, *Circle quantification*, *Candidate selection*, *Output*, and *Visualization using Decoil-viz*.

### Genome fragmentation

Decoil uses precomputed SV calls (VCF format) for the cycle reconstruction, which are computed in the paper using Sniffles 1.0.12 (–min_homo_af 0.7 –min_het_af 0.1 –min_length 50 –cluster –min_support 4) (Sedlazeck et al. 2018). The SV calls can also be provided as input by other equivalent tools, in VCF format. The SVs are filtered based on multiple criteria. Only SVs flagged as “PASS” or “STRANDBIAS,” having on target coverage ≥5× (default) and variant allele frequency (VAF) ≥ 0.01 (default), are kept. Breakpoints in a window size of 50 bp are merged. This curated breakpoints set *s* is used to segment the genome into *n* + 1 nonoverlapping fragments *f* ∈ *F*, where *F* represents the nonoverlapping fragments set.

### Graph encoding

The coverage and SV profiles were used to build a weighted undirected multigraph *G* = (*V*, *E*). A vertex *v* ∈ *V* represents either (1) the start (*t*, “tail”) or (2) the end (*h*, “head”) of a nonoverlapping genomic fragment object, with the property tuple (chromosome, position). An edge *e* ∈ *E* represents one of the three edge types: *e*_*f*_, *fragment edge*; *e*_*sv*_, *SV edge*; or *e*_*s*_, *spatial edge*. A nonoverlapping genomic fragment object *f* = (*t*, *h*, *e*_*f*_) ∈ *F* consists of a pair of two vertices, {“tail,” “head”} = {*t*, *h*}∈*V*, connected by a *fragment edge e*_*f*_  = (*t*, *h*, *w*_*f*_) ∈ *E*, with *w*_*f*_ as the weighted by the mean coverage spanning the genomic segment (Supplemental Fig. S10B). The two-node representation of *f* is used to track the orientation of the genomic fragment *f* ∈ *F* when traversing *G*. The edges *e*_*sv*_ ∈ *E* represent a SV connecting two fragments. The edges have two properties: (1) length defined as the SV length and (2) weight *w*_*sv*_ defined as *DR* (coverage of alternative variant). The SVs are encoded in the graph *G* based on their annotated type:

- BND, DEL—one edge connects “head” to “tail” of the two fragments;
- DUP—one edge connects “tail” to “head” of the two fragments;
- INV, INVDUP—two edges connect “head” to “head” and “tail” to “tail” of the two fragments; and
- Fragments with a mean coverage ≤5× (default) or standalone (*degree*(*v*) = 0) are discarded from the graph.

 The two fragments $f_{1}=(t_{1},h_{1},e_{\;f_{1}}),f_{2}=(t_{2},h_{2},e_{\;f_{2}})\in F$ are neighbors in the linear genomic space if *h*_1_ < *t*_2_ and are connected via *spatial edges e*_*s*_ = (*h*_1_, *t*_2_, *w*_*s*_) ∈ *E*, with *w*_*s*_ weight defined as the reads count spanning both *f*_1_, *f*_2_. A multigraph is used to represent scenarios when single fragment duplication occurs, that is, the fragment *f* = (*t*, *h*, *e*_*f*_) ∈ *F*, with *fragment edge e*_*f*_ = (*t*, *h*, *w*_*f*_) having an additional duplication edge *e*_*SV*_ (*SV edge*), connecting same two nodes {*t*, *h*}, *e*_*sv*_ = (*t*, *h*, *w*_*sv*_) (Supplemental Fig. S11).

### Search simple circles

Decoil searches all simple circular paths *c* = (*v*_1_, *v*_2_, …, *v*_*p*_) in the graph *G*, where *v*_*i*_ ∈ *V*, 1 ≤ *i* ≤ *p*, using weighted depth-first search (DFS) approach. A path in the DFS tree is circular if the end node *v*_*p*_ connects to any of the predecessor *v*_*i*_, 1 ≤ *i* ≤ *p* − 1 *backedge e* = (*v*_*i*_, *v*_*p*_). The weighted DFS is deterministic and guarantees a thorough exploration of circular paths across the entire genome. It achieves this by systematically traversing the tree structure, prioritizing edges based on their weights in a descending sorted order. The identified cycles are hashed and saved in a canonical form, in which the leftmost fragment corresponds to the 5′ leftmost genomic position. Duplicated cycles are removed during tree exploration. The resulting set comprises *unique simple cycles* (*S*). The simple cycles can share subpaths. The *simple cycles* set *S* is partitioned into *N* subsets, defined as partition *P* = {*M*_1_ …, *M*_*k*_, …, *M*_*N*_}, where *M*_*k*_ ∈ *P* is a subset that groups all simple cycles that share at least one genomic fragment.

### Circle quantification

This step filters artifacts and selects cycle candidates describing the amplification in the data. Because *P* is a partition of *S*, the subsets *M*_*k*_ ∈ *P* do not share genomic fragments, *k* index of *M*_*k*_, 1 ≤ *k* ≤ *N*. Therefore, the *Circle quantification* step (including the *LASSO* regression) was performed for each subset *M*_*k*_ individually. To allow the reconstruction of complex ecDNA structures, that is, large duplications and/or heavily rearranged, a *derived cycles* set (*D*_*k*_) was generated by computing all combinations between *simple cycles*. This step is combinatorial and therefore exponentially in size. In the real data set, an average of eight simple cycles per cluster were found by Decoil (Supplemental Fig. S9), which generates an input matrix of 256 rows for the *LASSO* regression and is computational feasible. However, cases with heavily rearranged genomic regions or small-deletion-dense regions can inflate exponentially the matrix size. Thus, filtering steps are applied to create a subset of $M_{k}^{*}$ that includes only sufficiently dissimilar simple cycles from *M*_*k*_ (see Supplemental Methods). Let *F*_*k*_ be the subset of all genomic fragments *F* that compose the *simple cycles M*_*k*_ and *derived cycles D*_*k*_. To find the parsimonious set of circular elements that describes the underlying coverage profile, a *LASSO* model was used to fit input features $X^{\left|F_{k}|\times (|M_{k}|+|D_{k}|\right)}$ against the targets $Y^{\left|F_{k}\right|}$, where *Y* = *X*β + β_0_, ${\text{β}}^{\left|M_{k}|+|D_{k}\right|}$ model coefficient vector. *LASSO* regularization generates a sparse solution; that is, it pulls model coefficients β to zeros, and it allows putative artifacts or cycle redundancies to be discarded. This means *LASSO* performs direct feature selection; that is, it selects a minimal set of likely cycle candidates. At the same time, it estimates the proportions of these cycles in the sample, which are the optimized coefficients β*. β_0_ is the intercept, estimated implicitly by *LASSO*, and it models the linear genome coverage to ensure a better estimation of the cycle proportions.

The optimization objective (cost function) for *LASSO* is (in line with the literature)

(1) E(β)+αR(β),

where *E*(β), the error term, is defined as

(2) $$E(\text{β})=\arg\underset{\text{β}}{\min}\left\{\frac{1}{\left|F_{k}\right|}\overset{\left|F_{k}\right|}{\underset{\;j=1}{\sum }}{\left(y_{j}-{\text{β}}_{0}-\overset{\left|M_{k}|+|D_{k}\right|}{\underset{i=1}{\sum }}x_{\;ji}{\text{β}}_{i}\right)}^{2}\right\}$$

and *R*(β), regularization term, is defined as

(3) $$R(\text{β})=\overset{\left|M_{k}|+|D_{k}\right|}{\underset{i=1}{\sum }}|{\text{β}}_{i}|.$$

 Let β* be the coefficients after the optimization (solution):

(4) $${\text{β}}^{*}=\mathrm{argmin}[E(\text{β})+\alpha R(\text{β})].$$

 To avoid overfitting of the model, a penalty term α = 0.1 was used. *x*_*ji*_ ∈ *X* is defined as the occurrence of fragment *f*_*j*_ in circle *c*_*i*_, with $c_{i}\in M_{k}\cup D_{k}$. *y*_*j*_ ∈ *Y* represents the mean coverage of the alignment spanning the genomic fragment *f*_*j*_. The optimized *LASSO* coefficients β* represent the estimated proportions of all cycles $c_{i}\in M_{k}\cup D_{k}$ (for an example, see Supplemental Fig. S10). In the final candidate cycle set *C*_*k*_, only *c*_*i*_ with a β_*i*_ > *t* was kept, where threshold *t* = max(min(*coverage*(*f*_*j*_)))/4. The higher the β_*i*_, the more likely is the cycle *c*_*i*_ to be a true ecDNA element. The final set contains all cycle candidates $C=\cup_{k=1}^{N}C_{k}$.

### Candidate selection

From the cycle candidate set, *C* was further reduced by filtering out cycles with estimated proportions β_i_ ≤ WGS mean coverage (default). Lastly, the circular elements >0.1 Mb (threshold published by Deshpande et al. 2019) are labeled as ecDNA, composing the cycle candidate set C*.

### Output

The algorithm outputs for the cycle candidate set C* the sequence in FASTA format and the reconstruction threads in BED-like format, which includes the information about (1) the mean coverage per fragment, (2) orientation of the fragment, (3) estimated proportions of circular element, and (4) the annotated topology (as defined in the paper). The *summary.txt* displays all found circular elements.

### Visualization using Decoil-viz

Lastly, for interpretability of the results, a visualization module was developed (https://github.com/madagiurgiu25/decoil-viz). This generates an HTML report to summarize all ecDNA reconstruction threads found by Decoil and to aggregate the information about the genomic fragments composing the amplicon, topology information, and estimated proportions. The implementation leverages gGnome (https://github.com/mskilab/gGnome), gTrack (https://github.com/mskilab-org/gTrack), and Rmarkdown (https://github.com/rstudio/rmarkdown).

### Ranking ecDNA topology definitions

To assess Decoil's reconstruction performance, we generated an in silico collection of ecDNA elements, spanning various sequence complexities for systematic evaluation. We introduced a ranking system and defined seven topologies of increasing computational complexity based on the SVs contained on the ecDNA element: (i) *Simple circularization*, there are no SVs on the ecDNA template; (ii) *Simple SVs*, ecDNA contains a series of either inversions or deletions; (iii) *Mixed SVs*, ecDNA has a combination of inversions and deletions; (iv) *Multiregion*, ecDNA contains different genomic regions from the same chromosome (DEL, INV, and TRA allowed); (v) *Multichromosomal*, ecDNA originates from multiple chromosomes (DEL, INV, and TRA allowed); (vi) *Duplications*, ecDNA contains duplications defined as a region >50 bp repeated on the amplicon (DUPs + other simple rearrangements); and (vii) *Foldbacks*, ecDNA contains a foldback defined as two consecutive fragments that overlap in the genomic space, with different orientations (INVDUPs + all other simple SVs). Every topology can contain a mixture of all other low-rank topologies.

### Simulate ecDNA

The simulation framework contains probabilistic variables, which model the chromosome weights, fragment position, fragment length, small deletion ratio, inversion ratio, foldback ratio, and tandem–duplication ratio. To cover a wide range of possible conformations, more than 2000 ecDNA sequence templates were generated. Based on these definitions, in silico ecDNA-containing samples were generated by simulating noisy long reads, at different depth of coverage, with an adapted version of PBSIM2 (Ono et al. 2021). This workflow is available at GitHub (https://github.com/madagiurgiu25/ecDNA-simulate-validate-pipeline). For a detailed description, see the Supplemental Methods.

### Performance evaluation on simulated data

To evaluate the correctness of reconstruction for Decoil, Shasta, and CReSIL, QUAST 5.2.0 (Mikheenko et al. 2018) was applied to compute different metrics (https://quast.sourceforge.net/docs/manual.html). Overall reconstruction performance was quantified as the mean and standard deviation of the largest contig metric. For a detailed description, see the Supplemental Methods.

### Evaluate amplicon's breakpoint recovery in ecDNA mixtures

To evaluate how well Decoil reconstructs ecDNA elements with overlapping genomic footprint, a series of dilutions was generated by mixing the CHP212, STA-NB-10DM, and TR14 cell lines at different ratios. Two types of mixtures were performed. First, 100% of one sample was combined with different percentages of another sample, that is, 10%, 25%, 50%, 75%, 90%, and 100% (Fig. 3C). Second, mixtures at different ratios for both samples were generated (10%–90%, 25%–75%, 50%–50%, 75%–25%, 90%–10%). Picard 2.26 (https://broadinstitute.github.io/picard/) was used to downsample the BAM files to 10%, 25%, 50%, 75%, and 90%, and SAMtools 1.9 (Li et al. 2009) was used to merge the different ratios and create in silico ecDNA mixtures. SV calling was performed using Sniffles 1.0.12 (Sedlazeck et al. 2018). Lastly, the ecDNA structures were reconstructed using Decoil using the mixture samples (BAM) and the SV profile. The ecDNA reconstructions were evaluated using as the metric the breakpoint recall/recovery, defined as the fraction of true breakpoints found in mixtures.

### Runtime and memory benchmarking

For both simulated and real data sets, we conducted an analysis of the runtime and memory usage. The runtime, including the raw elapsed time (ElapsedRaw) and CPU time (CPUTime), was measured. Additionally, memory usage was assessed using the maximum resident set size (MaxRss). These metrics were derived from the Slurm output, providing information about the computational resources consumed during the analysis.

### Ethics approval

Patients were registered and treated according to the trial protocols of the German Society of Pediatric Oncology and Hematology (GPOH). This study was conducted in accordance with the World Medical Association Declaration of Helsinki (2013) and good clinical practice; informed consent was obtained from all patients or their guardians. The collection and use of patient specimens were approved by the institutional review boards of Charité-Universitätsmedizin Berlin and the medical faculty of the University of Cologne. Specimens and clinical data were archived and made available by Charité-Universitätsmedizin Berlin or the National Neuroblastoma Biobank and Neuroblastoma Trial Registry (University Children's Hospital Cologne) of the GPOH. The *MYCN* gene copy number was determined as a routine diagnostic method using FISH.

### Software availability

Decoil is available freely as a docker and singularity container at GitHub (https://github.com/madagiurgiu25/decoil-pre). It can be run in two different ways: (1) Decoil-pipeline, a user-friendly Snakemake-workflow (Mölder et al. 2021), which takes as input a BAM file and computes internally the SV calling, the coverage profile, and ecDNA reconstruction, or (2) Decoil standalone, for more advanced and flexible usage, which requires as input a VCF file with the precomputed SV calling, a BW file with the coverage profile, and a BAM file. The visualization module, Decoil-viz, is freely available as a docker and singularity container at GitHub (https://github.com/madagiurgiu25/decoil-viz).

With this article, we publish several other associated tools and code repositories: a ecDNA sequence simulator based on specified topology (https://github.com/madagiurgiu25/ecDNA-sim), a long-read ecDNA containing samples simulator (adapted PBSIM2 for circular reference; https://github.com/madagiurgiu25/pbsim2), a Snakemake (Mölder et al. 2021) processing and validation pipeline for ecDNA containing simulated samples (https://github.com/madagiurgiu25/ecDNA-simulate-validate-pipeline), and the analysis associated with the paper available at GitHub (https://github.com/henssen-lab/decoil-paper) and at Zenodo (https://doi.org/10.5281/zenodo.10785693). Decoil, custom code, and all data are also available as Supplemental Code.

## Data access

All raw sequencing data for the patient samples and cell lines generated in this study have been submitted to the European Genome-phenome Archive (EGA; https://ega-archive.org) under accession numbers EGAS50000000348 and EGAS50000000349. Simulated ecDNA templates (BED) are available at Zenodo (https://doi.org/10.5281/zenodo.10785693).

## Supplementary Material

Supplement 1

Supplement 2

Supplement 3
