# Supplementary material for: Reconstructing extrachromosomal DNA structural heterogeneity from long-read sequencing data using Decoil
Source: Genome Res. 2024 Sep;34(9):1355–64. doi: 10.1101/gr.279123.124 (PMC11529853; doi:10.1101/gr.279123.124)
Supplement: Supplement 1 [file Supplemental_Materials.pdf]

Supplemental Materialsl

Supplemental Figures

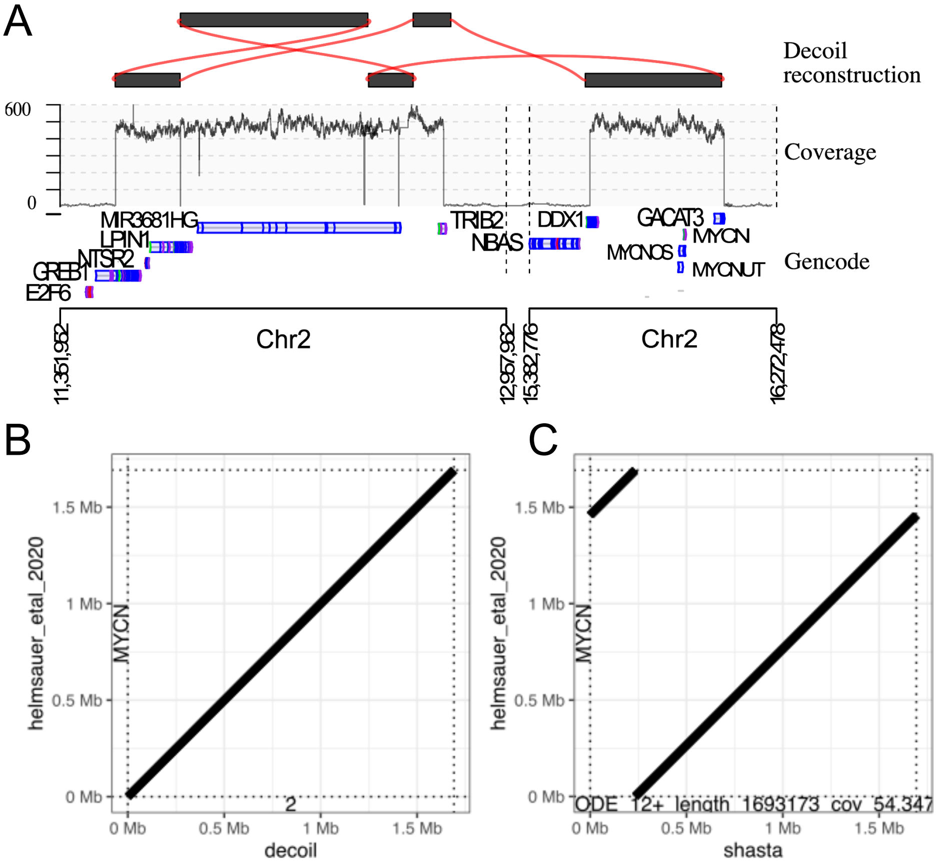

Supplemental Fig S1 CHP212 amplicon reconstruction. (A) Decoil reconstructs a Multi-region ecDNA structure. The tracks represent the reconstruction thread using Decoil-viz (top), coverage of the aligned reads (middle) and GENCODE v42 gene annotation (bottom). **(B)** Sequence identity comparison between Decoil (X-axis) and published coordinates Helmsauer et al. 2020 (Y-axis). **(C)** Sequence identity comparison between Shasta (X-axis) and published coordinates Helmsauer et al. 2020 (Y-axis).

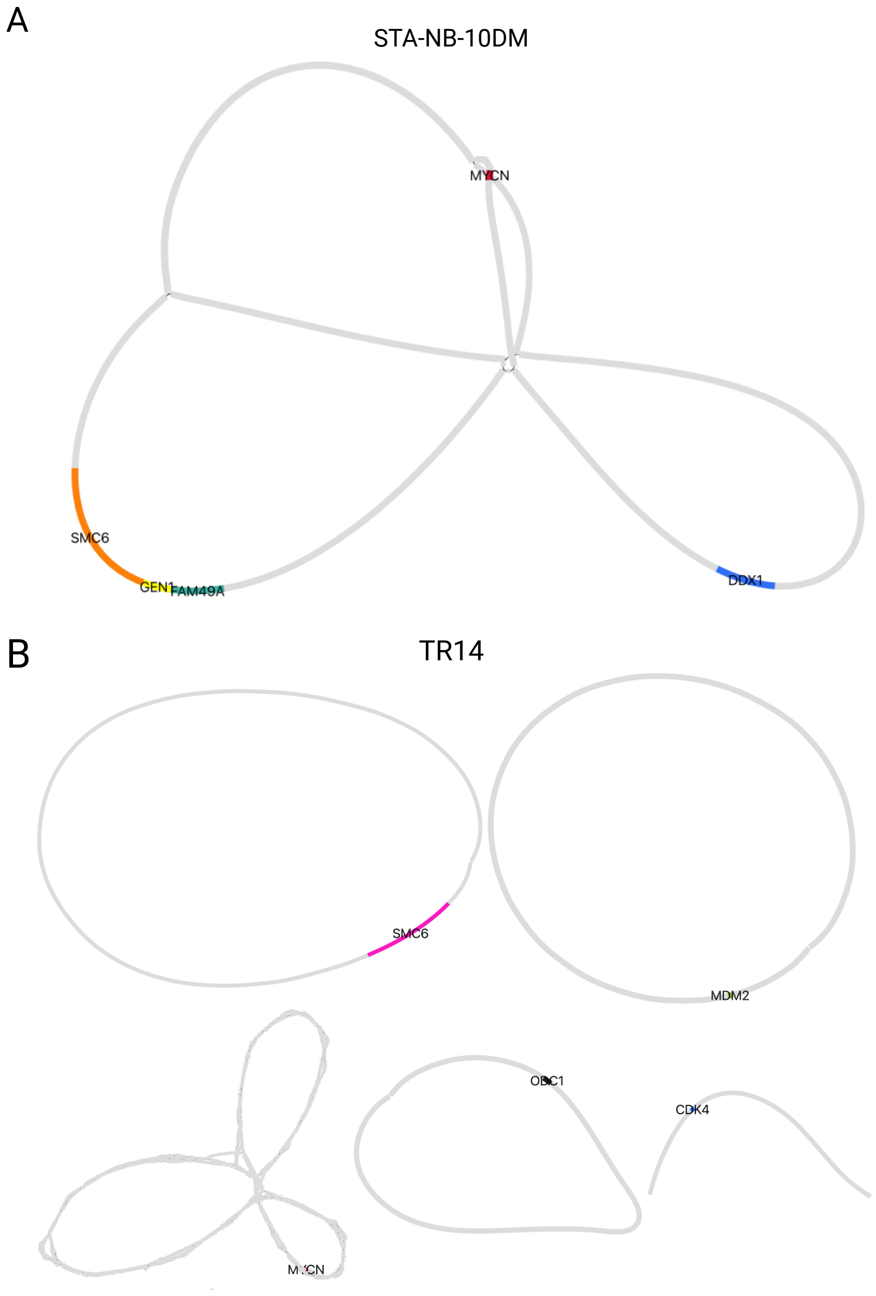

Supplemental Fig S2 Amplicons *de novo* assembly using Shasta. Bandage output showing the assemblies for all found ecDNA elements in two neuroblastoma cell lines. **(A)** STA-NB-10DM cell line containing one ecDNA element with co-amplification of *MYCN*, *DDX1*, *GEN1* and the fusion *SMC6-FAM49A*. Note that *SMC6* overlaps with *GEN1*. **(B)** TR14 cell line containing four circular assemblies *SMC6*, *ODC1*, *MDM2* and *MYCN*, whereas *CDK4* is resolved as a linear contig. *MYCN* locus is not a contiguous structure.

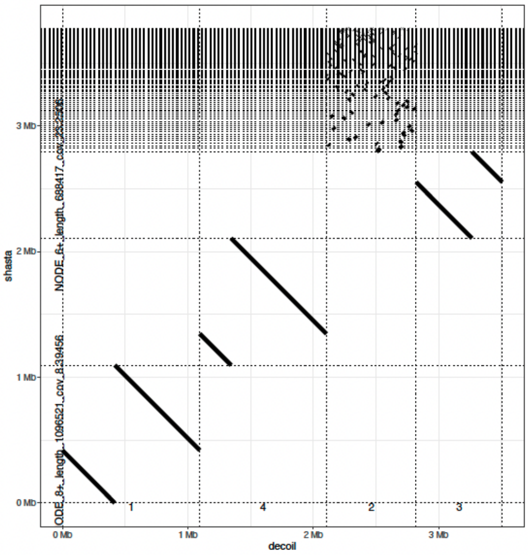

Supplemental Fig S3 Decoil and Shasta agreement for TR14 ecDNA elements. The dotplot shows the sequence identity between Shasta (Y-axis) and Decoil (X-axis). On the X-axis the numbers mean: 1 - *SMC6*, 4 - *MDM2*, 2 - *MYCN*, 3 - *ODC1* amplicon.

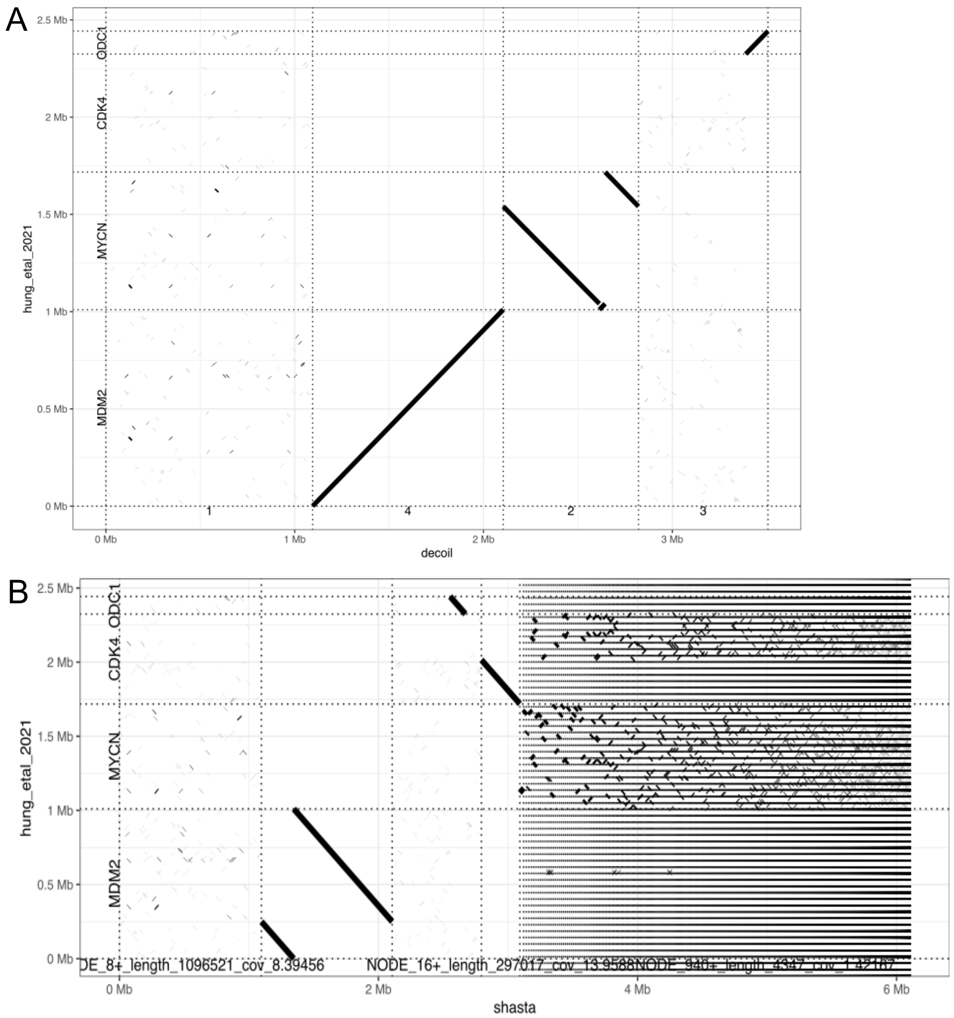

Supplemental Fig S4 Sequence identity of reconstructions and published amplicons. Comparison between Decoil **(A)** and Shasta **(B)** reconstructions (X-axis) and Hung et al. 2021 (Y-axis) for the TR14 ecDNA elements.

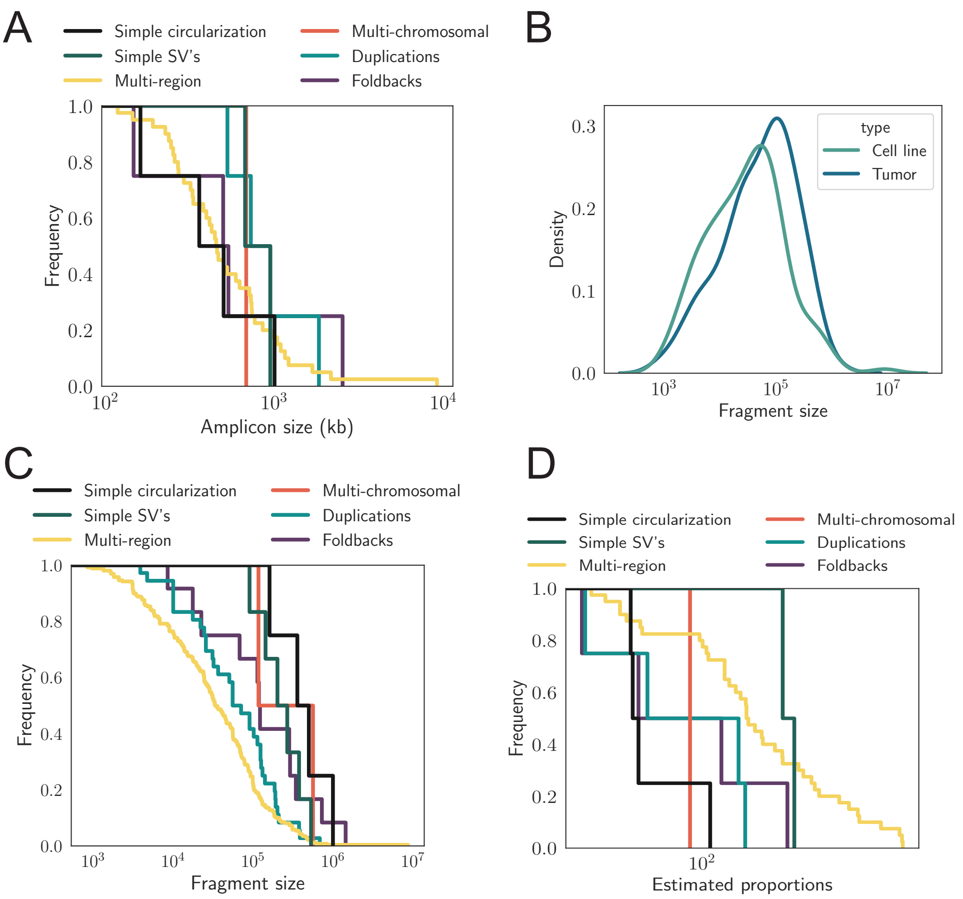

Supplemental Fig S5 ecDNA amplicon features (extended). **(A)** Frequency (Y-axis) of the amplicon size (X-axis) for the identified ecDNA topologies across cell lines and patient samples. **(B)** Fragment size (X-axis) distribution (Y-axis) of the reconstructed amplicons. **(C)** Fragment size (X-axis) frequency. **(D)** Frequency of the estimated proportions of the amplicons across the different identified topologies. The colors in **(C,D)** corresponds to the legend in **(A)**.

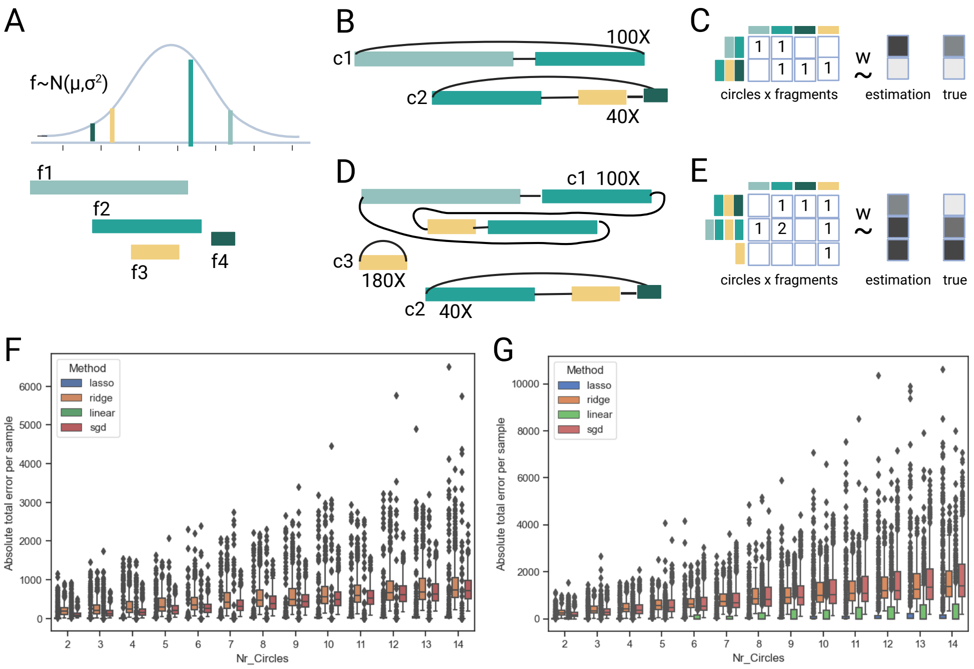

Supplemental Fig S6 Comparison of linear models to deconvolve ecDNA elements from simulated overlapping fragments data. **(A)** Fragments $f1-4$ lengths are sampled from a normal distribution $N(7000, 3000)$. **(B)** ecDNA elements examples ($c1$,$c2$) with overlapping fragments and amplicons copies of 100× and 40×. $c1$ and $c2$ contain unique fragments. **(D)** Complex scenario of ecDNA elements with overlapping fragments. $c1$ contains a duplicated fragment (turquoise) and $c3$ is structurally a subcycle of $c1$ and $c2$. **(C,E)** Numerical values of the *LASSO* regression input matrix for the ecDNA structure examples in **(B,D)**. **(F,G)** Performance evaluation of four regression models, i.e. *LASSO* (blue), Ridge (orange), Linear regression (green) and SGD (red), for selecting and estimating correctly the proportions of the ecDNA structures overlapping in the genomic space in simulated data. X-axis represents the number of simulated ecDNA elements per sample and Y-axis quantifies the absolute total error distribution of the regression models fit. Lower values mean low error. Boxplots show Q1 (25%), Q2 (median) and Q3 (75%), interquartile range IQR = Q3 - Q1, and whiskers are 1.5×IQR. (See Extended Methods). Created with BioRender.com.

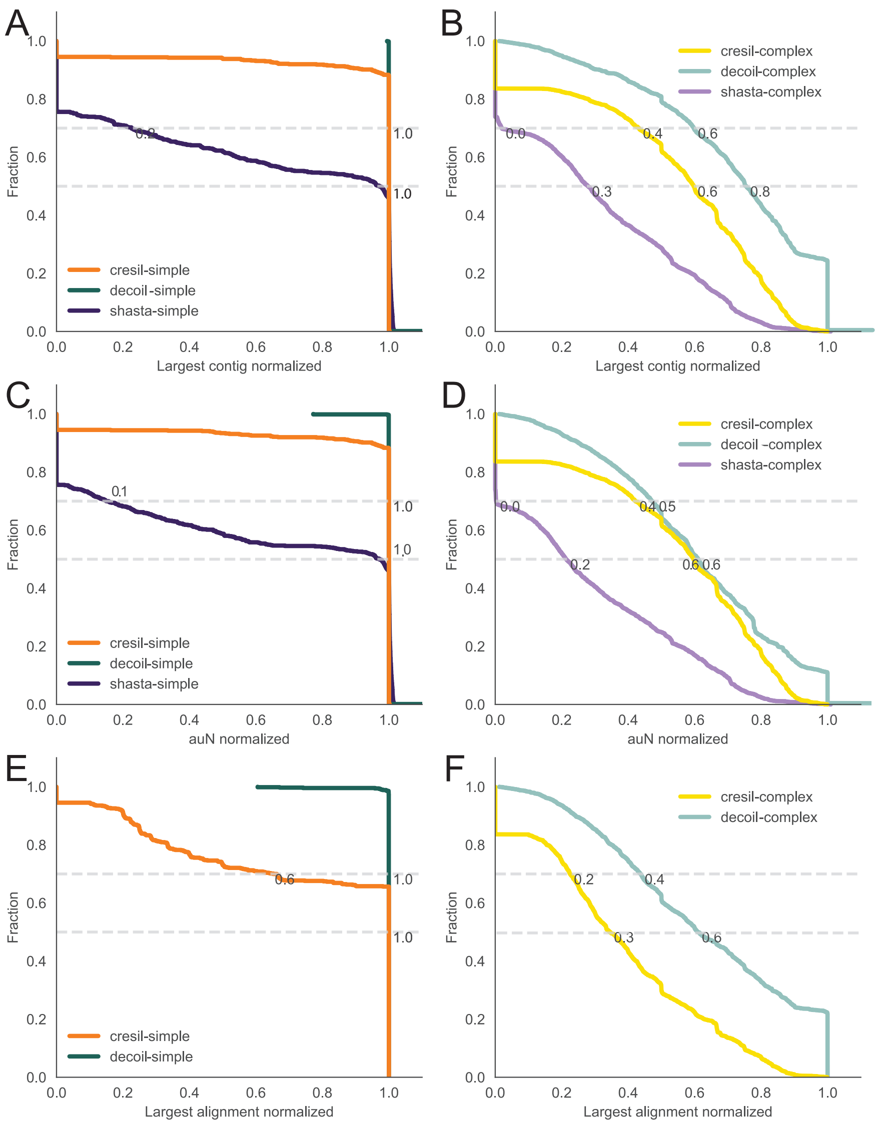

Supplemental Fig S7 Decoil, Shasta and CReSIL comparison for the simple and complex ecDNA structures for > 2000 simulations using different metrics. Cummulative curve of the largest contig, auN and largest alignment for simple **(A,C,E)** and complex **(B,D,F)** ecDNA simulations. **(A,B)** X-axis represents the largest contig normalized by the true structure length (1 - a good reconstruction, 0 - poor reconstruction, values > 1 refer to reconstructions larger than the true structure). Y-axis shows the fraction of reconstructions with the specific contiguity for the three methods: Decoil (dark green / light green), CReSIL (orange / yellow), Shasta (dark purple / light purple). **(C,D)** X-axis represents the auN (area under N50) normalized by the true structure length. **(E,F)** X-axis represents the largest alignment normalized by the true structure length. This metric was available only for Decoil and CReSIL. The gray horizontal lines are at 0.5 and 0.7 fraction in all panels.

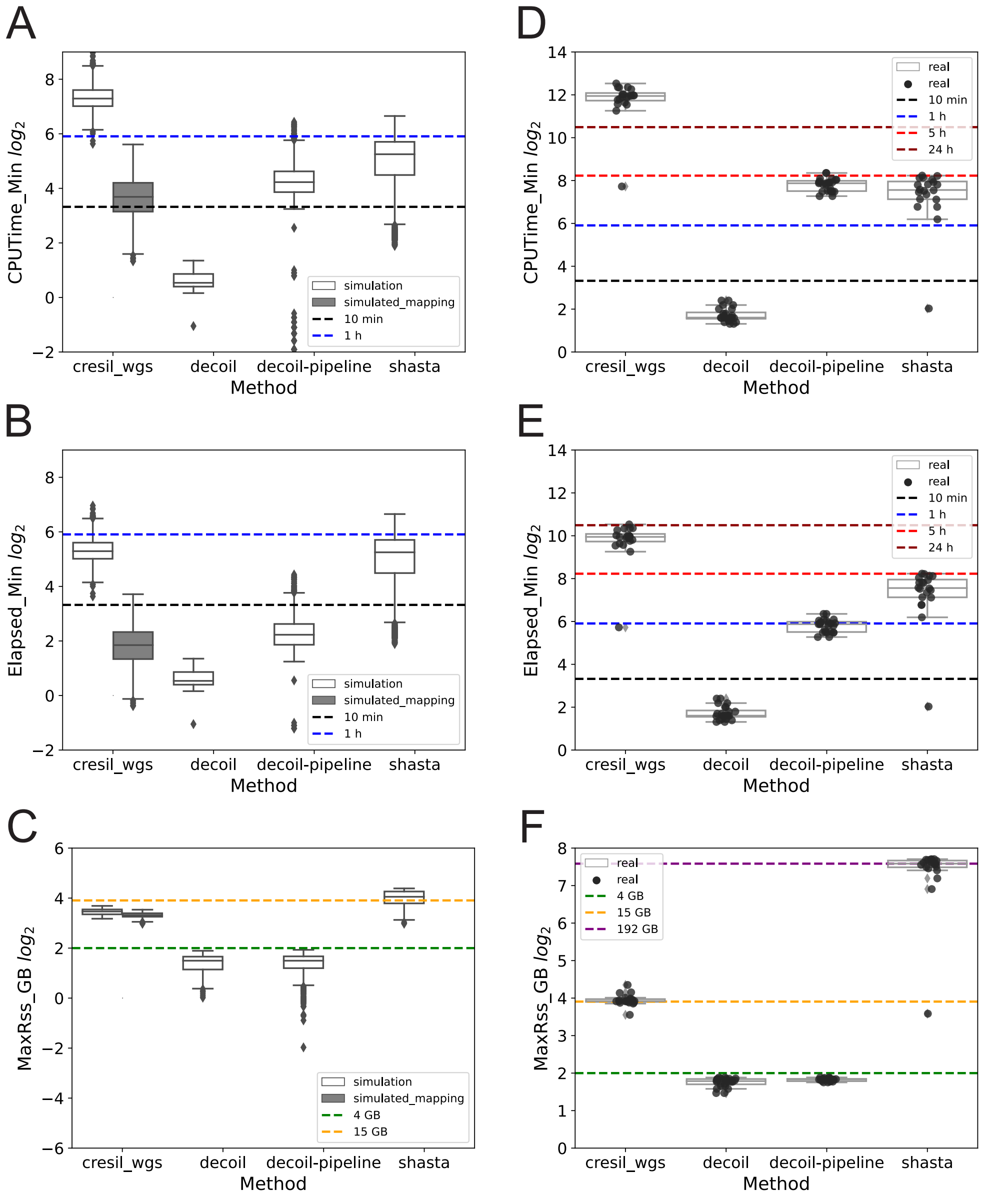

Supplemental Fig S8 Runtime and memory benchmark for the simulated and real datasets. **(A)** CPUTime (log_2(min)) , **(B)** Elapsed time / User time (log_2(min)) and **(C)** maximum used memory in log_2(GB) for the simulated data (> 2000 data points). **(D)** CPUTime (log_2(min)), **(E)** Elapsed time / User time (log_2(min)) and (F) maximum used memory (MaxRSS) in log_2(GB) for real data, i.e. shallow long-read whole-genome sequencing data (3-7× mean coverage, > 20 data points). Shasta takes as input a FASTQ. CReSIL takes as input a FASTQ and performs internally alignment using minimap2 (in orange). Decoil starts with the SV calling, coverage track precomputed. Decoil-pipeline takes as input a BAM file and computes internally SV calling and the coverage track. For CReSIL, Decoil, Decoil-pipeline 4×threads were used, for Shasta only 1×thread due to intensive memory usage. The boxplot shows Q1 (25%), Q2 (median) and Q3 (75%), interquartile range IQR = Q3 - Q1, and whiskers are 1.5×IQR.

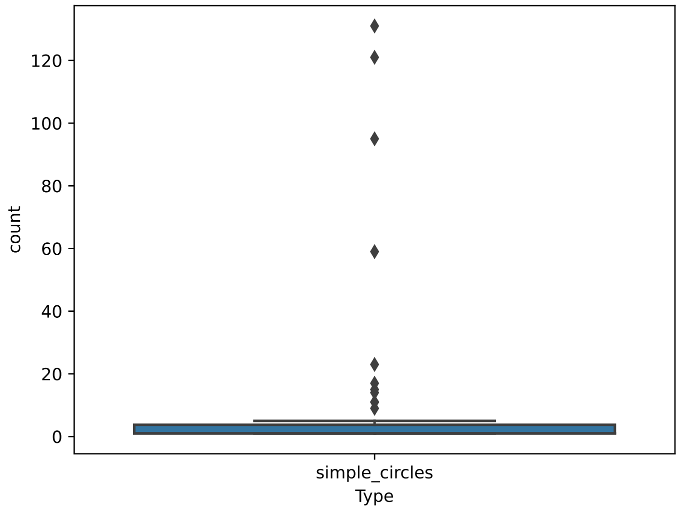

Supplemental Fig S9 Overlapping simple cycles per cluster distribution. Y-axis (count) represents the number of simple cycles which cluster together for the real sequencing dataset, for the *MYCN* locus, i.e. have at least one overlapping fragment (mean = 8.9 and median = 1). The boxplot shows Q1 (25%), Q2 (median) and Q3 (75%), interquartile range IQR = Q3 - Q1, and whiskers are 1.5×IQR.

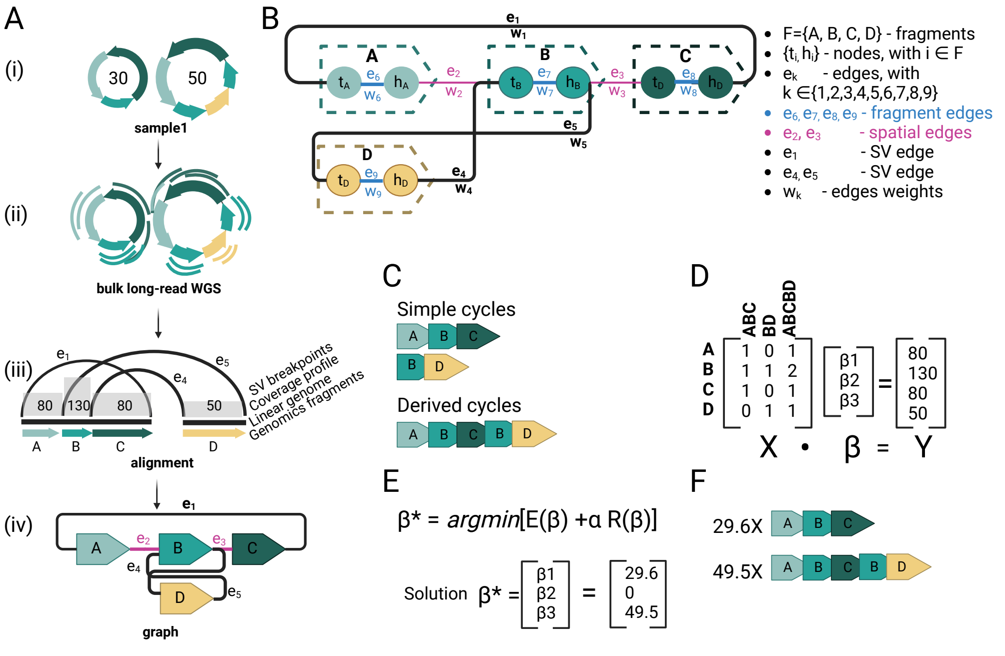

Supplemental Fig S10 Decoil graph encoding and the numerical values of the Lasso regression applied on a toy example. **(A)** A toy example **sample1** contains two ecDNA structure (i) with overlapping genomic regions and copy numbers 30 (left), 50 (right). This is sequenced using bulk long-read WGS (ii) and generates specific SV and coverage profiles (iii). The coverage profile spans the genomic fragments A (light green), B (turquoise), C (dark green), D (yellow) with a mean coverage across the individual segments of approximately 80, 130, 80 and 50×. The structural variants profile consists of the following SV's: e_1 (DUP), e_4 (BND) and e_5 (BND). (iv) The graph encodes as nodes the genomic fragments objects $F = \{A, B, C, D\}$ and as edges $e_k \in E = \{e_1, e_2, e_3, e_4, e_5\}$, where e_1, e_4, e_5 are *SV edges* and e_2, e_3 (pink) are *spatial edges*. A *spatial edge* connects two fragments which are neighboring in the genomic space. **(B)** The graph implementation of the SV and coverage profiles. Each genomic fragment $i \in F$ is represented in the graph as a pair of nodes (t_i, h_i) connected by a *fragment edge* (blue). For example fragment A is composed of (t_A, h_A) and connected by e_6 (blue). The weights w_6, w_7, w_8, w_9 (blue) represent the mean coverage spanning the genomic fragments A, B, C, D. The weights w_2, w_3 (pink) correspond to the *spatial edges* e_2, e_3 and represent the number of reads spanning the neighboring fragments. The weights w_1, w_4, w_5 (black) corresponds to the *SV edges* e_1, e_4, e_5 and represent the supporting reads of the called SV. **(C)** Decoil discovers in this example two simple cycles (ABC, BD) and one derived cycle (ABCBD). **(D)** The numerical values of the input features X and output target Y for the *LASSO* regression, for **sample1**. The target Y vector represents the mean coverage across the fragments A, B, C, D. The coefficients β are estimated by *LASSO*. (E) The cost function for *LASSO* regression to be optimized (see Methods), with $E(\beta)$ error term, $R(\beta)$ regularization term, β^* best model, and the solution of the estimated coefficients β by *LASSO* regression. α set to 0.1. **(F)** The selected ecDNA structures based on the β coefficients, i.e. ABC, ABCBD and their estimated proportion, i.e. 29.6 and 49.5. Created with BioRender.com.

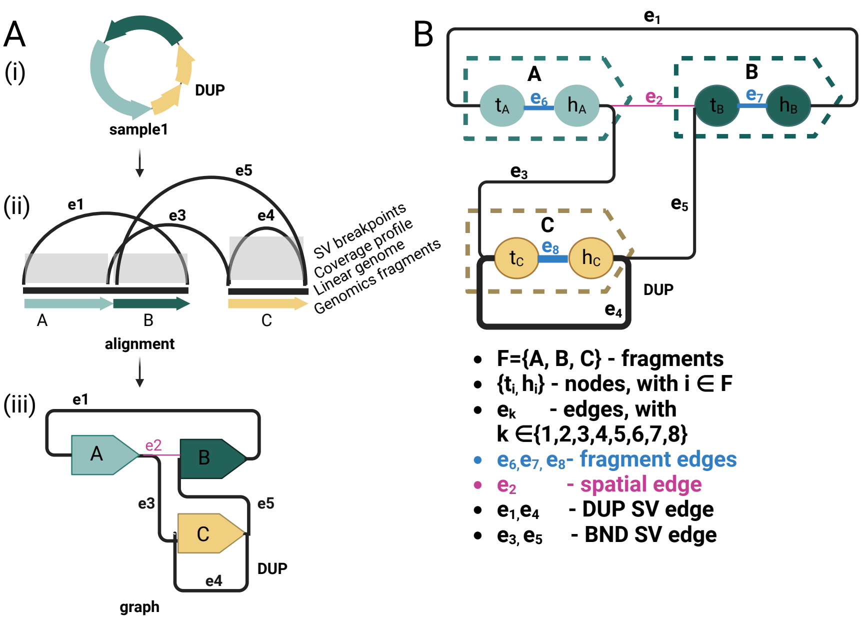

Supplemental Fig S11 Multigraph example. **(A)** ecDNA structure containing a duplication (yellow segment) (i), which is composed of four different SVs (e1,e3,e4,e5) (ii). These SV variants are encoded in the graph (iii) and connect the genomic fragment A, B, C. **(B)** The underlying multigraph, with the collection of nodes $V = \{t_A, h_A, t_B, h_B, t_C, h_C\}$, edges $e_k \in E = \{e_1, e_2, e_3, e_4, e_5, e_6, e_7, e_8\}$ and genomic fragment objects $F = \{A, B, C\}$. Each genomic fragment object is composed of two nodes, a head and a tail. E.g. fragment C is composed of the nodes t_c (tail), h_c (head), connected by a *fragment edge* e_8 (blue). C is duplicated on the ecDNA and this variation is encoded as edge e_4 (black). Thus, two edges, e_8 and e_4, connect the same nodes two t_c, h_c, exemplifying why a multigraph is required. Created with BioRender.com.

Supplemental Tables

Supplemental Table S1 Performance evaluation for Decoil, Shasta and CReSIL. Every value in the table represents the mean and standard deviation for the entire simulated dataset. All the metrics are normalised by the true length of the simulated ecDNA. 1 means correct assembly, <1 assembly regions were missed, >1 assembly regions were additional included.

Assembly contiguity	auN_norm		Largest_contig_norm	
Topology	Simple	Complex	Simple	Complex
Decoil	1.00 ± 0.01	0.65 ± 0.55	1.00 ± 0.01	0.76 ± 0.59
Shasta	0.80 ± 0.32	0.37 ± 0.24	0.82 ± 0.29	0.41 ± 0.22
CReSIL	0.92 ± 0.23	0.52 ± 0.28	0.93 ± 0.23	0.52 ± 0.28

Supplemental Table S2 Assembly contiguity for Decoil, Shasta and CReSIL in simulated data. The metrics are computed using QUAST. All the metrics are normalised by the true length of the simulated ecDNA. For Largest_contig_norm, Total_length_norm, N50_norm, auN_norm, Largest_alignment_norm (not available for Shasta), Total_aligned_length_norm 1 means correct assembly, <1 assembly regions were missed, >1 assembly regions were additional included. The Misassembled_contigs_length_norm shows how much (fraction) of the assembled output diverge from the reference/true structure. Unaligned_length_norm represents genomic region fraction missed by the reconstruction or assembly (see Supplemental Methods for the full definition of these metrics).

	Shasta	Decoil	CReSIL
Largest_contig_norm	0.53 ± 0.31	0.82 ± 0.54	0.74 ± 0.23
Total_length_norm	0.91 ± 0.99	1.03 ± 0.61	0.75 ± 0.22
N50_norm	0.52 ± 0.33	0.8 ± 0.54	0.74 ± 0.23
N90_norm	0.43 ± 0.38	0.51 ± 0.6	0.73 ± 0.24
auN_norm	0.49 ± 0.33	0.74 ± 0.52	0.74 ± 0.23
Misassembled_contigs_length_norm	0.05 ± 0.19	0.23 ± 0.37	0.36 ± 0.39
Unaligned_length_norm	0.48 ± 1.09	0.02 ± 0.47	0.0 ± 0.0
Largest_alignment_norm	/	0.73 ± 0.29	0.56 ± 0.3
Total_aligned_length_norm	0.49 ± 0.35	1.0 ± 0.34	0.74 ± 0.22

Supplemental Table S3 Features of the reconstructed amplicons for cell lines. Total size (Mb), estimated proportions and topology of the reconstruction, computed by Decoil.

Cell line	Amplicon	Size (Mb)	Estimated proportions	Topology
CHP212	*MYCN*	1.69	440	Multi-region
STA-NB-10DM	*MYCN*	2.1	253	Multi-region
TR14	*SMC6*	1.09	20	Multi-chromosomal
TR14	*MDM2*	1.01	109	Simple circularization
TR14	*MYCN*	0.7	487	Multi-chromosomal
TR14	*ODC1*	0.68	89	Multi-chromosomal

Supplemental Table S4 ecDNA elements reconstruction description in patients by Decoil.

Patient	ID	Size Mb	Estimated proportions	Topology
Patient1	E1	0.9	226	Simple SV's
Patient2	E2	1.09	44	Multi-region
Patient3	E3	2.55	122	Foldbacks
Patient3	E4	0.3	50	Simple circularization
Patient4	E5	0.22	732	Multi-region
Patient4	E6	0.39	756	Multi-region
Patient5	E7	0.5	53	Foldbacks
Patient5	E8	0.77	47	Multi-region
Patient5	E9	0.41	55	Multi-region

Supplemental Table S5 Median runtime and memory for simulated and real datasets. Elapsed time and CPUTime are given in minutes. MaxRss is given in GB. Decoil and Shasta are single threaded. Decoil-pipeline and CReSIL were run using 4 × threads. CReSIL (mapping) represents the runtime and memory quantification for the mapping step, internally called by CReSIL. CReSIL (total) represents the total time.

Dataset	Simulated			Real		
Measure	Elapsed	CPUTime	MaxRss	Elapsed	CPUTime	MaxRss
Decoil	1.45	1.45	2.8	3.04	3.04	3.4
Decoil-pipeline	4.68	18.73	2.8	58.61	243.46	3.5
CReSIL (mapping)	3.6	12.88	9.89	na	na	na
CReSIL (total)	39.16	156.55	11.04	986.54	3946.16	15.13
Shasta	38.06	38.06	16.59	188.7	188.7	192.12
