# Supplementary material for: Reconstructing extrachromosomal DNA structural heterogeneity from long-read sequencing data using Decoil
Source: Genome Res. 2024 Sep;34(9):1355–64. doi: 10.1101/gr.279123.124 (PMC11529853; doi:10.1101/gr.279123.124)
Supplement: Supplement 3 [file Supplemental_Methods.pdf]

Supplemental Methods

This document comprises an extension to the Methods section from the main manuscript and the supplementary information aims to increase readability of the manuscript.

DNA extraction and nanopore sequencing

High molecular weight (HMW) DNA was extracted from 5 to 10 million cells or 15 to 25 mg of tissue using the MagAttract HMW DNA kit (Qiagen N.V., Venlo, Netherlands) according to the manufacturer's protocol. DNA concentration was measured with a Qubit 3.0 Fluorometer (Thermo Fisher) and quality control was performed using a 4200 TapeStation System (Agilent Technologies, Inc., Santa Clara, CA). For library preparation, the Ligation Sequencing Kit (SQK-LSK109 or SQK-LSK110, Oxford Nanopore Technologies Ltd, Oxford, UK) was used. All libraries were sequenced on a R9.4.1 MinION flowcell (FLO-MIN106, Oxford Nanopore Technologies Ltd, Oxford, UK) for more than 24 h.

Decoil algorithm

Decoil (deconvolve extrachromosomal circular DNA isoforms from long-read data) is a graph-based method to reconstruct circular DNA variants from shallow long-read WGS data. This uses (1) structural variants (SV) and (2) focal amplification information to reconstruct circular ecDNA elements. The algorithm consists of seven modules: *Genome fragmentation*, *Graph encoding*, *Search simple circles*, *Circles quantification*, *Candidates selection*, *Output* and *Visualization using Decoil-viz*. All the modules are fully described in the main Methods section of the manuscript, except for *Circles quantification* module, which is below described.

Circles quantification

This steps filters artifacts and quantifies the likely cycles describing the amplification in the data. Because P is a partition of S, the subsets $M_k \in P$ do not share genomic fragments, k index of M_k, $1 <= k <= N$. Therefore, the circle quantification step (including the $LASSO$ regression) was performed for each subset individually. To allow the reconstruction of complex ecDNA structures, i.e. large duplications and/or heavily rearranged, a *derived cycles* set (D_k) was generated, by merging/combining *simple cycles*, which share a genomic region. In the real dataset an average of 8 simple cycles per cluster were found by Decoil (**Supplemental Fig S9**), which generates an input matrix of 256 rows for the $LASSO$ regression and is computational feasible. However, small deletions and very rearranged genomes can inflate the matrix exponentially and discover many simple cycles with high sequence identity per subset/cluster (M_k). Therefore only *simple cycles* sufficiently dissimilar are considered. For this purpose, a filtered subset M_k^* was computed, by excluding *simple cycles* with an similarity higher equal than $>= x$ (default 0.9) (keep the longer cycles). The similarity was defined as the jaccard index JC:

$$JC = \frac{F_1 \cap F_2}{F_1 \cup F_2} = \frac{\sum length(f_i)}{\sum length(f_j)} \tag{5}$$

Where

- $F1, F2$ - fragment sets describing the cycles $c_1, c_2 \in M_k$
- $length(f_i), length(f_j)$, length of fragments f_i, f_j
- Fragments $f_i \in F_1 \cap F_2$, $f_j \in F_1 \cup F_2$

Using the filtered subset M_k^*, the *derived cycles* set D_k was created by performing all the combinations, in the mathematical sense, e.g. $\binom{|M_k^*|}{2}$, $\binom{|M_k^*|}{3}$, ..., between *simple cycles* $c \in M_k^*$, which are sufficiently dissimilar in the fragment composition with $JC <= s_{max}$, (default 0.7). Which means, per subset M_k^* there can be at maximum

$2^{|M_k^*|} - 1 - |M_k^*|$ combinations.

Example: Let S=c1,c2,c3,c4 be all simple cycles and P=M1, M2 be the partition of S, where every subset, M1=c1,c2,c3 and M2=c4 contains simple cycles with overlapping region. For M1 we compute all derived cycles, by combining all simple cycles in M1, i.e D1 = c1c2,c1c3,c2c3. From M2 we cannot compute derived cycles. We assume that c1,c2,c3 have a pair-wise $JC <= s_{max}$.

Let F_k be the subset of all genomic fragments F which compose the *simple cycles* M_k and *derived cycles* D_k. To find the parsimonious set of circular elements which describes the underlying coverage profile, a *LASSO* model was used to fit input features $X^{|F_k| \times (|M_k|+|D_k|)}$ against the targets $Y^{|F_k|}$, where $Y = X\beta + \beta_0$, $\beta^{|M_k|+|D_k|}$ model coefficients vector. *LASSO* regularization generates a sparse solution, i.e. it pulls model coefficients β to zeros and it allows putative artifacts or cycles redundancies to be discarded. This means, *LASSO* performs direct feature selection, i.e. it selects a minimal set of likely cycles candidates. At the same time, it estimates the proportions of these cycles in the sample, which are the optimized coefficients β^*. β_0 is the intercept, estimated implicitly by *LASSO*, and, it models the linear genome coverage to ensure a better estimation of the cycles proportions.

The optimization objective (cost function) for *LASSO* is (in line with the literature):

$$E(\beta) + \alpha R(\beta) \quad (6)$$

Where, $E(\beta)$, error term, defined as:

$$E(\beta) = \arg\min_{\beta} \left\{ \frac{1}{|F_k|} \sum_{j=1}^{|F_k|} \left(y_j - \beta_0 - \sum_{i=1}^{|M_k|+|D_k|} x_{ji}\beta_i \right)^2 \right\} \quad (7)$$

And $R(\beta)$ regularization term, defined as:

$$R(\beta) = \sum_{i=1}^{|M_k|+|D_k|} |\beta_i| \tag{8}$$

Let β^* be the coefficients after the optimization (solution):

$$\beta^* = \arg\min[E(\beta) + \alpha R(\beta)] \tag{9}$$

To avoid overfitting of the model, a penalty term $\alpha = 0.1$ was used. $x_{ji} \in X$ is defined as the occurrence of fragment f_j in circle c_i, with $c_i \in M_k \cup D_k$. $y_j \in Y$ represents the mean coverage of the alignment spanning the genomic fragment f_j. The optimized *LASSO* coefficients β^* represent the estimated proportions all cycles $c_i \in M_k \cup D_k$ (example in **Supplemental Fig S10**). In the final candidates cycles set C_k, only c_i with a $\beta_i > t$ were kept, where threshold $t = max(min(coverage(f_j))/4$. The higher the β_i the more likely is the cycle c_i to be a true ecDNA element. The final set contains all cycles candidates $C = \cup_{k=1}^{N} C_k$.

Ranking system of ecDNA topologies

To assess Decoil's reconstruction performance, we generated an *in-silico* collection of ecDNA elements, spanning various sequence complexities for systematic evaluation. We introduced a ranking system and defined seven topologies of increasing computational complexity, based on the SV's contained on the ecDNA element:

1. *Simple circularization* - no structural variants on the ecDNA

2. *Simple SV's* - ecDNA element contains either a series of inversions or deletions
3. *Mixed SV's* - ecDNA element has a combination of inversions and deletions
4. *Multi-region* - ecDNA element contains different genomic regions from the same chromosome (DEL, INV and TRA allowed)
5. *Multi-chromosomal* - ecDNA element originates from multiple chromosomes (DEL, INV and TRA allowed)
6. *Duplications* - ecDNA element contains duplications defined as a region larger than 50 bp repeated on the amplicon (DUP's + other simple rearrangements)
7. *Foldbacks* - ecDNA element contains a foldback defined as a two consecutive fragments which overlap in the genomic space, with different orientations (INVDUP's + all other simple SV's)

Every topology can contain a mixture of all other low-rank topologies.

Simulate ecDNA sequence templates

The simulation framework contains probabilistic variables, which model the chromosome weights, fragment position, fragment length, small deletion ratio, inversion ratio, foldback ratio, and tandem-duplication ratio. Simulation strategy for individual ecDNA templates starts by choosing the genomic position relative to previous simulated fragment, covering four scenarios (**Fig 2A#1**):

- neighbor - the next simulated fragment starts right next to the previous fragment
- [0 to 5 kb] - the next simulated fragment starts within a 5 kb distance relative to the previous fragment
- >5 kb - the next simulated fragment starts a least at a 5 kb distance relative to the previous fragment
- switch chromosome - the next simulated fragment is sampled from another chromosome

Next, to simulate small deletions (DELs), $< 10\%$ of fragment size can be cut out with a certain probability p, at the left or right end of the fragment (Fig 2a#2). With a probability p, inversions (Fig 2a#3) and tandem-duplications (Fig 2a#4) are simulated. To cover a wide range of possible conformations we generate first a so-called conformation array, which encodes the different event types for describing the simulation of individual ecDNA template. The conformation array has binary entries (except the first position which encodes the fragments number), where every bit is set to 0 (disable the occurrence of the event on ecDNA) or to 1 (allows the occurrence of the event with a probability p).

Supplementary Table S6 Conformation array for ecDNA templates simulation. N_FRAG - number of fragments; SMALL_DEL - allow small deletions on the right and left side of the fragment; DUP - allow simple duplication; INV - allow inversions; INTERCHR - allow fragments to originate from multiple chromosomes; MULTI_REGION - allow fragments to originate from multiple regions on same chromosome; FOLDBACK - allow foldbacks, which are here defined as two overlapping genomic fragments, which are immediately chained in the ecDNA template, regardless of the strand orientation.

N_FRAG	SMALL_DEL	DUP	INV	INTERCHR	MULTI_REGION	FOLDBACK

Conformation array for the seven topologies based on which multiple rounds of simulations were performed:

Simple circularization:

1	0	0	0	0	0	0

Simple SV's:

2 - 10	[0|1]	0	0	0	0	0

Mixed SV's:

2 - 10	1	0	1	0	0	0

Multi-region :

2 - 10	[1|0]	0	[1|0]	0	1	0

Multi-chromosomal:

2- 10	[1|0]	0	[1|0]	1	1	0

Duplications:

2 - 10	[1|0]	1	[1|0]	[1|0]	[1|0]	0

Foldbacks:

2 - 10	[1|0]	[1|0]	[1|0]	[1|0]	[1|0]	1

In total 577 conformation arrays were obtained, based on which more than 2000 ecDNA templates were generated. Code available under https://github.com/madagiurgiu25/ecDNA-sim.

Simulate *in-silico* long-read ecDNA-containing samples

To assess ecDNA reconstruction performance, *in-silico* ecDNA-containing samples were generated based on the ecDNA sequence templates collection. The workflow takes as input the defined ecDNA elements in BED format and generates its associated FASTA reference. Afterwards, noisy long-reads, with an average length of 7,000 bp, are sampled from this reference using an adapted version of PBSIM2 (Ono et al. 2021 Ono et al. (2021)), at a specified depths of coverage. This package was customized for the purpose of this paper to (1) allow reads sampling from a circular reference, and (2) provide a better coverage uniformity of the reads at fragments boundary by using Mersenne twister (Harase 2014 Harase (2014)) instead of the pseudorandom number generator included in the original package (https://github.com/madagiurgiu25/pbsim2). The *in-silico* reads are stored in FASTQ format. This workflow steps is part of the benchmarking pipeline https://github.com/madagiurgiu25/ecDNA-simulate-validate-pipeline.

Alignment-free ecDNA reconstruction using Shasta from simulated data

To *de novo* assemble the simulated ecDNA the reads were filtered using NanoFilt De Coster et al. (2018) 2.6.0 (-l 300 -q 20 –headcrop 20 –tailcrop 20). *De novo* assembly was performed using Shasta Shafin et al. (2020) 0.10.0 with parameters –config Nanopore-May2022 –Reads.minReadLength 1000 –Kmers.distanceThreshold 500 –Kmers.probability 0.5.

Alignment-based ecDNA reconstruction using Decoil from simulated data

To reconstruct the ecDNA using Decoil, the reads were filtered using NanoFilt De Coster et al. (2018) 2.6.0 (-l 300 -q 20 –headcrop 20 –tailcrop 20), aligned to the reference genome GRCh38/hg38 using ngmlr Sedlazeck et al. (2018) 0.2.7 with stardard parameters. Structural variant calling was performed using sniffles Sedlazeck et al. (2018) 1.0.12 (–min_homo_af 0.7 –min_het_af 0.1 –min_length 50 –cluster –min_support 4) and the bigWig coverage tracks were computed using bamCoverage (-50 bins) from deepTools Ramírez et al. (2016) 3.5.1 suite. Decoil used the alignment, SV calls and coverage profile as input to reconstruct simulated ecDNA (–min-vaf 0.01 –min-cov-alt 6 –min-cov 8 –max-explog-threshold 0.01 –fragment-min-cov 10 –fragment-min-size 500).

Alignment-based ecDNA reconstruction using CReSIL from simulated data

Simulated ecDNA was also identified using CReSIL Wanchai et al. (2022) v1.0.0 [https://github.com/visanuwan/cresil, commit:646aec9], with standard parameters, using ‘cresil trim‘, followed by ‘cresil identify_wgls‘ and reference genome GRCh38/hg38.

Performance evaluation on simulated data for Decoil, Shasta and CReSIL

For benchmarking purpose, Decoil was compared against two additional methods, CReSIL, a long-read method for reconstructing small and large circular elements, and Shasta, a *de novo* assembler. Decoil and CReSIL are alignment-based methods, whereas Shasta is a alignment-free approach.

CReSIL uses a graph-based approach, similarly to Decoil, to discover cycles. CReSIL implements a directed multi-graph, where a node represents a genomic fragment (including orientation) and an edge is a linkage connecting two regions. CReSIL starts with raw reads (FASTQ), whereas Decoil starts with a BAM file and pre-computed SV calls. Decoil implements an undirected multi-graph, where nodes are the genomic fragments start / ends sites (to track fragment orientation) and SV supporting reads are edges. This allows traversing the genomic fragments in both forward and reverse orientation, which allows the discovery of inverted duplications, which is not possible if the node has a predefined orientation, as in the case of CReSIL. Decoil merges simple cycles into derived cycles to allow the discovery of complex rearrangements, containing e.g. large duplications. CReSIL searches for the longest path in the subgraph covering all nodes/genomic-fragments, approach which cannot resolve duplications or foldbacks on the amplicon. Additionally, Decoil uses a *LASSO* model to select likely cycles, by removing putative artifacts and redundancies. This approach allows to discover confidently co-occurring ecDNA structures with shared genomic loci on the amplicon, which is not possible with CReSIL method. The challenge of resolving the structure of co-occurring ecDNA structures with shared genomic loci, i.e. deconvolving ecDNA structure heterogeneity from bulk WGS data, was not previously addressed by any method from bulk WGS and represents a significant

improvement over state-of-the art methods.

To evaluate the accuracy of ecDNA reconstructions, QUAST Mikheenko et al. (2018) 5.2.0 was applied to compute different metrics (https://quast.sourceforge.net/docs/manual.html). The overall reconstruction performance was quantified as the mean and standard deviation of the largest contig metric, defined as the longest contig in the assembly. The contiguity of the reconstruction was visualized using dotplots, for which paf alignments from the true and reconstructed were generated using minimap2 Li (2021) 2.26-r1175.

Metrics definitions used for the comparison (adapted from QUAST):

- Largest_contig_norm - Largest contig is the length of the longest contig in the assembly, normalized by true length
- Total_length_norm - is the total number of bases in the assembly, normalized by true length
- N50_norm - is the length for which the collection of all contigs of that length or longer covers at least half an assembly, normalized by true length
- N90_norm - same as N50 but but with 90% instead of 50%
- auN_norm - is the area under the N50, normalized by true length. This metric was proposed and justified by Heng Li in his blog https://lh3.github.io/2020/04/08/a-new-metric-on-assembly-contiguity.
- Largest_alignment_norm - is the length of the largest continuous alignment in the assembly, normalized by true length
- Total_aligned_length_norm - is the total number of aligned bases in the assembly, normalized by true length
- Misassembled_contigs_length_norm - is the total number of bases in misassembled contigs, normalized by true length

Linear models comparison to deconvolve ecDNA elements from simulated overlapping fragments data

In order to identify the probable ecDNA components within a given sample, we employ a regression-based technique to deconvolve the circular paths that best align with the coverage profile and determine their estimate proportions. Four different regression models were tested, i.e *LASSO* (Least Absolute Shrinkage and Selection Operator), Rigde regression, Linear regression and SGD (Stochastic Gradient Descent) regression. This experiment simulates the matrix formulation for the regression. In the simulation it can be specified how many fragments two different circular structure share and randomly chosen. The amplicon copies per circular structure is sampled from a normal distribution $\mathcal{N}(200, 150)$. For every fragment of the circular the length is samples from a normal distribution $\mathcal{N}(7000, 3000)$. To calculate the error between predicted coverage Y_p and true coverage profile Y_t the total absolute error was used, i.e. $e = \sum_i |y_{pi} - y_{ti}|$, with $y_{pi} \in Y_p$ and $y_{ti} \in Y_t$.

Evaluate amplicon's breakpoints recovery in ecDNA mixtures

To evaluate how well Decoil can reconstruct ecDNA elements with overlapping footprints a series of dilutions was generated by mixing the CHP212, STA-NB-10DM and TR14 cell lines at different ratios. We generated two types of mixtures. First, 100% of one sample with different percentages of another sample, i.e. 10, 25, 50, 75, 90, 100% **(Fig 3C)** were combined. Secondly, mixtures at different ratios for both samples (10-90, 25-75, 50-50, 75-25, 90-10%) were generated. Picard 2.26 (https://broadinstitute.github.io/picard/) was used to downsample the BAM files to 10, 25, 50, 75, 90% and SAMtools 1.9 to merge the different ratios and to create *in-silico* ecDNA mixtures. SV calling was performed using sniffles Sedlazeck et al. (2018) 1.0.12 with same parameters as for the original 100% BAM files, i.e. –min_homo_af 0.7 –min_het_af 0.1 –min_length 50 –cluster –min_support 4. Decoil was run on all these

mixtures with parameters –min-vaf 0.01 –min-cov-alt 10 –min-cov 10 –max-explog-threshold 0.01 –fragment-min-cov 10 –fragment-min-size 500. The completeness of the reconstructed ecDNA elements in mixtures was evaluated by counting how many breakpoints are identical compared to the true ecDNA elements in the 100% samples.

Preprocess nanopore sequencing data from cell lines and patient samples

For the analysis five neuroblastoma cell lines and 13 patients were sequenced using shallow whole-genome sequencing. For all the samples the status of *MYCN* amplification on ecDNA was experimentally determined by FISH. The patients cohort included 10 patients ecDNA positive and three ecDNA negative, serving as negative control. One ecDNA-containing sample was removed from the analysis due to failed QC. The cell lines CHP212, TR14, STA-NB-10DM and all 13 patient samples were preprocessed by performing base-calling using Guppy 5.0.14 (dna_r9.4.1_450bps_hac model), followed by a quality check using NanoPlot 1.38.1. The reads were filtered by quality using NanoFilt De Coster et al. (2018) 2.8.0 (-l 300 –headcrop 50 –tailcrop 50) and aligned using ngmlr Sedlazeck et al. (2018) 0.2.7 against the reference genome GRCh38/hg38. The structural variant calling was performed using sniffles Sedlazeck et al. (2018) 1.0.12 (–min_homo_af 0.7 –min_het_af 0.1 –min_length 50 –min_support 4). The bigWig coverage tracks were obtained by applying bamCoverage (-50 bins) from deepTools Ramírez et al. (2016) 3.5.1 suite. The cell lines LAN-5 and CHP126 were similarly processed using the reference genome GRCh37/hg19. The pipeline is available under https://github.com/henssen-lab/nano-wgs.

Reconstruct ecDNA elements for cell lines and patient samples using Decoil

To reconstruct the ecDNA elements for CHP212, TR14 and STA-NB-10DM Decoil was applied using the parameters –min-vaf 0.1 –min-cov-alt 10 –min-cov 8 –fragment-min-cov 10 –fragment-min-size 1000 –filter-score 35 or –min-vaf 0.01 –min-cov-alt 10 –min-cov 10 –max-explog-threshold 0.01 –fragment-min-cov 10 –fragment-min-size 500, the reference genome GRCh38/hg38 and annotation GENCODE V42. Similarly, for LAN-5 and CHP126 the ecDNA reconstruction was performed using Decoil with same parameters, reference genome GRCh19/hg19 and annotation GENCODE V41. The ecDNA elements in patient samples were reconstructed by Decoil using –min-vaf 0.1 –min-cov-alt 10 –min-cov 30 –max-explog-threshold 0.01 –fragment-min-cov 20 –fragment-min-size 100.
