# Supplementary material for: Reconstructing extrachromosomal DNA structural heterogeneity from long-read sequencing data using Decoil
Source: Genome Res. 2024 Sep;34(9):1355–64. doi: 10.1101/gr.279123.124 (PMC11529853; doi:10.1101/gr.279123.124)
Supplement: Supplement 2 [file Supplemental_Code.zip › genome_research_decoil_code/ecDNA-simulate-validate-pipeline-1.0.1.zip!/ecDNA-simulate-validate-pipeline-1.0.1/dag_simplified.pdf]

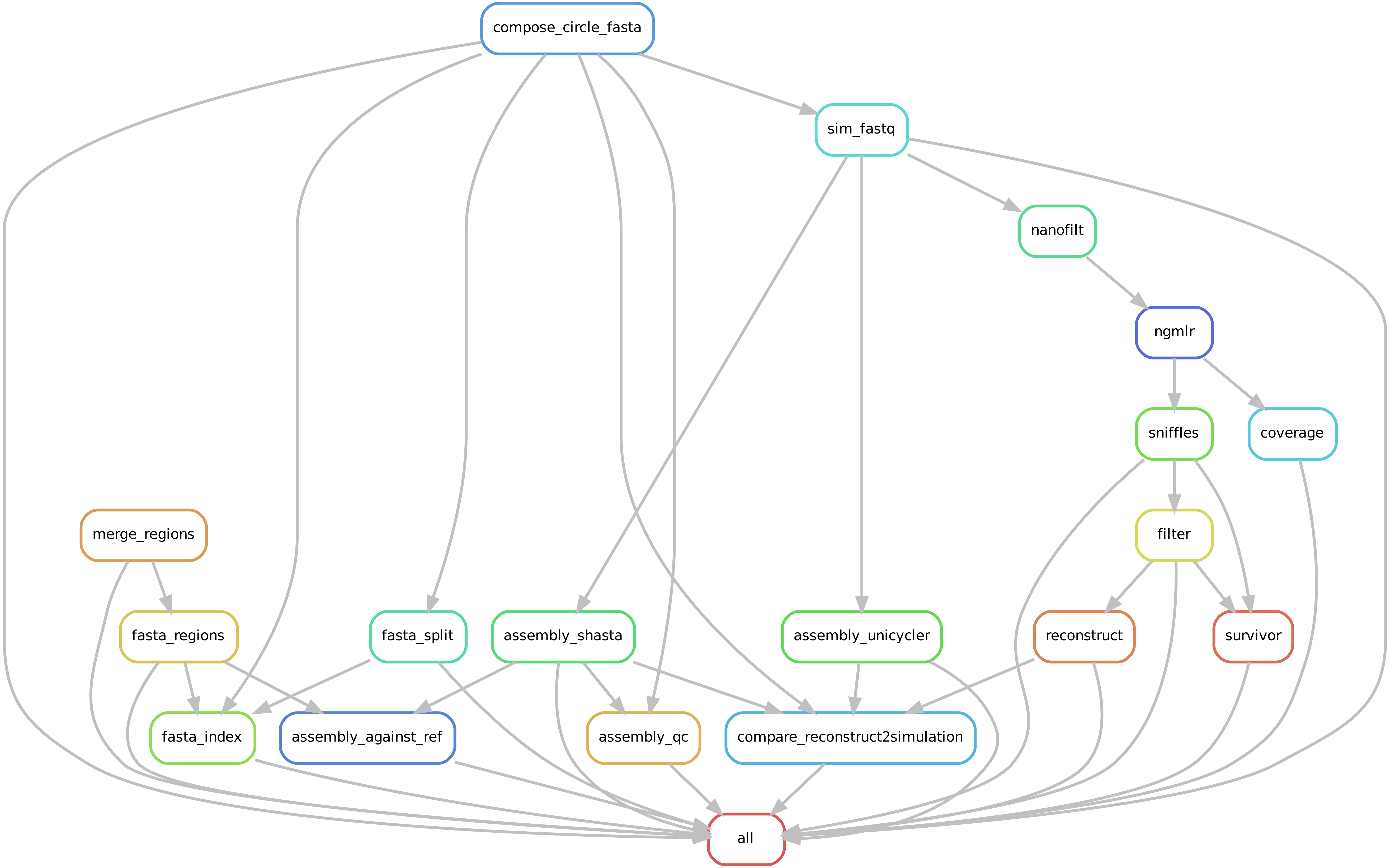
compose_circle_fasta
sim_fastq
nanofilt
ngmlr
sniffles
coverage
merge_regions
filter
fasta_regions
fasta_split
assembly_shasta
assembly_unicycler
reconstruct
survivor
fasta_index
assembly_against_ref
assembly_qc
compare_reconstruct2simulation
all
